# A Novel Intrauterine Device for the Extended Tissue-Specific Release of Estradiol and Norethindrone to Treat the Genitourinary Syndrome of Menopause

**DOI:** 10.3390/polym17050665

**Published:** 2025-02-28

**Authors:** Ahmed Abdelgader, Mershen Govender, Pradeep Kumar, Yahya E. Choonara

**Affiliations:** Wits Advanced Drug Delivery Platform Research Unit, Department of Pharmacy and Pharmacology, School of Therapeutic Sciences, Faculty of Health Sciences, University of the Witwatersrand, Johannesburg, 7 York Road, Parktown, Johannesburg 2193, South Africapradeep.kumar@wits.ac.za (P.K.)

**Keywords:** genitourinary syndrome of menopause, controlled release, estradiol, ethyl cellulose, intrauterine device, norethindrone acetate, polycaprolactone

## Abstract

The genitourinary syndrome of menopause (GSM) is a prevalent condition impacting a substantial number of women globally. Presently, the management of GSM typically entails the administration of estrogen via oral, dermal, or vaginal routes for a prolonged period of time. This study involves the development of a polymer-based hollow cylindrical delivery system loaded with estradiol hemihydrate (E2) for prolonged delivery to the uterine cavity (EPHCD) combined with a norethindrone acetate (NETA)-loaded polymeric matrix (NLPM), with both units placed onto an intra-uterine device to form a multi-component drug delivery system for the management of GSM (MCDDS). In developing EPHCD, a central composite design (CCD) was employed to evaluate and optimize the impact of formulation factors on EPHCD release and unit weight loss. The optimized EPHCD was further assessed for its chemical integrity, surface morphology, hydration characteristics, release behavior, ex vivo permeation and cytocompatibility. The optimized EPHCD, which featured a high drug load (10%) and low ethyl cellulose-to-polycaprolactone ratio (EC-to-PCL, 10%), demonstrated favorable attributes with a cumulative drug release and weight loss of 23.78 ± 0.84% and 2.09 ± 0.21%, respectively, over a 4-week testing period. The release kinetics were further noted to obey the Peppas–Sahlin model. Evaluation of MCDDS revealed an in vitro drug release comparable to the individual units, with permeation studies displaying an initial increase in the rate of flux for both drugs during the first 2 h, followed by a subsequent decrease. Moreover, the MCDDS components showed good cytocompatibility against NIH/3T3 cells, with cell viability of more than 70%. Upon evaluation of the MCDDS system, the results of this study highlight its potential as a viable sustained-release intrauterine platform for the treatment of GSM.

## 1. Introduction

The innovation of novel formulations and delivery modalities for existing drugs presents significant opportunities to extend the product lifespan and ameliorate the financial landscape of the pharmaceutical industry [1]. Recently, substantial attention has been directed towards implantable drug delivery systems (IDDSs) incorporating polymeric biomaterials [2,3,4]. These systems serve as a platform for the extended release of therapeutic agents, from weeks to months, and in some instances, extending to years. The primary objective of these systems is to maintain therapeutic drug concentrations within a desirable range. Moreover, IDDSs serve as an instrumental strategy for localized drug administration, ensuring heightened drug concentrations at specific anatomical sites, while minimizing systemic exposure and consequent adverse effects [5,6,7,8,9,10,11].

GSM is a prevalent condition caused by diminished estrogen level, mainly during menopause, and its management requires an extended treatment regimen encompassing the duration of the condition [12]. Estrogens are commonly administered either as monotherapy or in combination with progestins to relieve the symptoms of severe and moderate menopause [13,14,15,16]. However, due to the severe side effects and risks associated with estrogen use, which depend on the duration of time and amount to be used, the lowest efficient dose is recommended when long-term regimen treatment is indicated [15,16].

A variety of estrogen-marketed medications are viable to address GSM, administered through various routes and dosage forms [17,18,19]. These formulations include vaginal tablets (10 μg of estradiol/tablet), which are intended for daily use over 2 weeks, followed by 2 tablets per week as a maintenance dose. Estrace cream (0.01% estradiol) is applied daily for 2 weeks, followed by a maintenance dose of 1 to 3 times weekly. Additionally, Imvexxy softgel capsules (4 μg or 10 μg per insert), as well as Estring^®^ and Femring^®^ intravaginal rings, are also employed, available with different estradiol loads [12]. These delivery systems, however, have shown numerous shortfalls, directly influencing patient acceptability. The most common associated with these systems include frequent dosing, a short retention time, messiness, bleeding, discharge, and breast pain [12].

**Figure 1:**
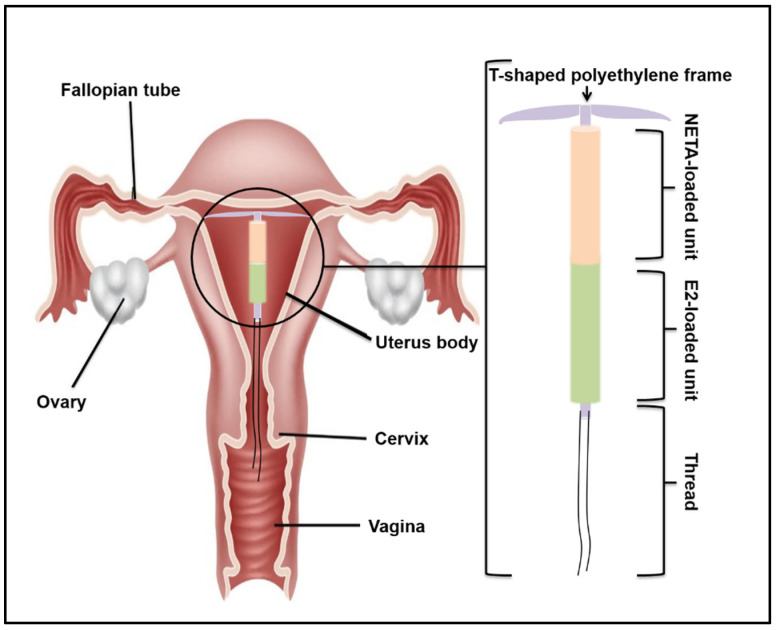
Schematic representation depicting the E2 and NETA components placed on the T-shaped frame, forming the MCDDS. The figure was adapted from Abdelgader et al. [21], © 2024 by the authors, Licensee MDPI, Basel, Switzerland.

An effective strategic approach to address GSM and its currently marketed medication challenges involves the development of intrauterine implantable delivery systems (IUDs) capable of sustaining hormone release over an extended duration. In alignment with these concepts, this study has developed a system comprising an estradiol hemihydrate-loaded hollow cylindrical device (EPHCD), prepared using a polycaprolactone (PCL) and ethyl cellulose (EC) blend, which aims to deliver a pharmacologically effective dose of estradiol hemihydrate (E2) into the uterine cavity over an extended duration, thus minimizing the frequency of dosing. The anticipated advantages encompass prolonged therapeutic effects, improved cost-effectiveness, and enhanced patient compliance [20]. The fabrication of EPHCD was achieved via the solvent-assisted melting technique, with a rotatable CCD employed to optimize formulation parameters, such as drug load and polymer ratio (EC-to-PCL percentage), to achieve the desired degradation and drug release kinetics profiles. Subsequently, the optimized EPHCD underwent further characterization studies to assess its morphology, hydration capacity, and chemical integrity. Once the optimized EPHCD was developed, it was combined with a norethindrone acetate-loaded polymeric matrix (NLPM) on a 3D-printed IUD, as described in our previous work [21], to form an MCDDS, to overcome the risks associated with long-term use of estrogenic monotherapy, such as endometrial hyperplasia and the risk of endometrial cancer [22]. Drug release studies, drug permeation assessments using uterine tissue, and cytocompatibility evaluations have further been undertaken to evaluate the potential of MCDDS for the treatment of GSM. Figure 1 illustrates the schematic architecture of the MCDDS intrauterine delivery system, showcasing the integration of the two-drug-loaded platforms designed to release E2 and norethindrone acetate (NETA).

## 2. Materials and Methods

### 2.1. Materials

Estradiol hemihydrate (purity: 98.4%) and norethindrone acetate (purity: 99.2%) were procured from Leap Chem (Hong Kong, China), while ethyl cellulose and polycaprolactone (PCL; Mn  =  80,000) were purchased from Sigma-Aldrich Chemie GmbH (St. Louis, MO, USA) and Sigma-Aldrich (Tokyo, Japan), respectively. Mouse fibroblast cells (NIH/3T3; ATCC CRL-1658) were sourced from American Type Culture Collection (ATCC, Manassas, VA, USA). Dulbecco’s Modified Eagle Medium (DMEM) was acquired from Life Technologies Limited (Paisley, UK), fetal bovine serum from PAN Biotech (Aidenbach, Germany), and 3-(4,5-dimethylthiazol)-2,5-diphenyl-tetrazolium bromide (MTT) solution along with solubilizing buffer from Roche Diagnostic GmbH (Mannheim, Germany). All other chemicals employed were of analytical grade and were utilized without further purification.

### 2.2. Methods

#### 2.2.1. Preparation and Statistical Optimization of EPHCD

EPHCDs were fabricated utilizing a custom-designed mold comprising a 1 mL propylene syringe assembly, as detailed in our previously published work [21]. Briefly, polymer mixtures (30% *w/v*) were prepared by dissolving varying ratios of polymers in acetone heated to 45 °C, with EC added first, followed by PCL after 6 min. E2 powder (5, 7.5, or 10% *w/w*) was thereafter incorporated into the molten polymer blend after 25 min and thoroughly mixed. The drug–polymer mixtures were subsequently loaded into the customized mold and then quickly cooled at −80 °C for 4 h to facilitate the crystallization of polymers. Thereafter, the solidified segments were demolded, stored overnight at −80 °C, and dried under ambient conditions for 72 h. Once dried, all segments were inspected for surface defects, cut to the desired length, capped with a 5% PCL solution, and stored at 20 °C for further analysis.

The impact of varying levels of independent formulation variables on key attributes of EPHCD, thus determining optimized combinations, was assessed and explored utilizing Design Expert^®^ V8 software (StatEase, Inc., Minneapolis, MN, USA). For this, a rotatable CCD template with α = 1.414 was used to produce EPHCD formulations involving combinations of PCL, EC, and E2. Two formulation variables, E2 load (X_1_) ranging from 5% to 10%, and the ratio of EC-to-PCL (X_2_) ranging from 10% to 50%, were chosen for the design of experiments. The effects of these variables were studied on key EPHCD outputs, including drug release percentages at weekly intervals over 4 weeks (Y1, Y2, Y3, and Y4, respectively) and the degree of weight loss after incubation in simulated uterine fluid (SUF) for 8 weeks (Y5). Specifically, drug load was evaluated as part of the CCD due to its impact on drug release (Y1–Y4) and its influence on the device weight loss profile.

Fourteen experimental runs (EH1–EH14) were conducted, as outlined in Table 1. In order to facilitate comparative analyses, control formulations devoid of the drug (DF) were prepared in correspondence to the E2-loaded formulations (Table 1). The optimal mathematical models were selected based on an assessment of statistical parameters, such as *p*-value, R^2^, adjusted R^2^, predicted R^2^, CV, and predicted residual sum of squares (PRESS). The polynomial equation of 2nd order was established to express the relationship between responses and input parameters and is presented as follows:

(1) $$Y=b_{0}+b_{1}X_{1}+b_{2}X_{2}+b_{12}X_{1}X_{2}+b_{11}X_{1}^{2}+b_{22}X_{2}^{2}$$

where *X*_1_, *X*_2_ represent the main effects, $X_{1}^{2}$ and $X_{2}^{2}$ denote the quadratic effects, and *X*_1_*X*_2_ signify the interaction effect. The term *Y* corresponds to the responses, while *b*_0_ is the intercept representing the arithmetic mean of all quantitative outcomes. The terms *b*_1_, *b*_2_, *b*_12_, *b*_11_, *b*_22_ are the regression coefficients associated with the respective effects of each variable.

The 3D and contour plots were generated from these equations to visualize the impact of formulation factors on measured responses. Response optimization was further performed using numerical and graphical techniques, employing the desirability approach and overlay plots, respectively, to achieve an optimized solution with desirable drug load and polymeric proportions.

##### Determination of the Unit Diameter and Drug Content Uniformity

The measurement of the diameter for each individual EPHCD was performed employing a digital vernier caliper. In addition, an assessment of percent content uniformity was conducted, to ascertain accurate dose delivery and ensure homogeneous drug distribution throughout the device. This assessment was performed using a method previously reported in the literature [23,24], with minor modifications. In brief, samples (n = 3; 2 mm length) from each EPHCD design formulation were sliced, weighed, and dissolved in 2 mL acetonitrile within a 40 °C oil bath. Thereafter, 8 mL of methanol was added to the resultant mixture, followed by centrifugation at 12,000 rpm for 10 min. The supernatant was then analyzed spectrophotometrically at 280 nm (ε = 0.0073; R^2^ = 0.998) with the drug content thereafter calculated.

##### Degradation in SUF and Alkaline Conditions

Specimens (n = 3) of 5 mm EPHCD with a specific initial weight (*W_i_*) were assessed for their biodegradation in SUF (pH 7; 15 mL) by incubating the samples in polytops retained in a shaker (25 rpm, 37 °C), with degradation media replenished every week. After 8 weeks, the specimens were removed, rinsed with purified water, and then air-dried until constant weight (*W_f_*) was achieved. Similarly, the method reported by Boia et al. [25] was adapted to assess the different EPHCD and corresponding DF formulations’ degradation under alkaline conditions. Briefly, EPHCD and drug-free device samples (n = 3) of 2 mm length were retained overnight in purified water. Thereafter, the EPHCD specimens were dipped in 3 mL of 5 M NaOH solution in sealed polytops for 10 min, then gently wiped with filter paper to eliminate surface moisture, prior to being accurately weighed (*W_i_*). The weighed samples were then returned to 10 mL NaOH. At predetermined times of 4, 24, 48, and 96 h, the samples were extracted, the excess moisture removed as previously described, prior to being accurately weighed (*W_f_*). Degradation and assisted degradation at high pH, described in terms of weight loss percentage, were thereafter calculated using Equation (2). The method reported by Yang and co-workers was used to prepare the SUF [26].

(2) $$\%\;Weight\;loss=\frac{\left(W_{i}-W_{f}\right)}{W_{i}}\times 100$$

##### Textural Analysis

Physico-mechanical characteristics of the EPHCD and DF formulations, particularly their hardness, were examined through the determination of resistance to indentation employing a ball probe of 5 mm attached to a textural analyzer (TA.XT.plus; Stable Micro Systems, Surrey, UK), and following the methodology reported previously [21]. In brief, samples of 4 mm diameter and 5 mm length (n = 3) were mounted horizontally and pressed to a depth of 0.3 mm. The maximum force generated from the applied force over the sample surface was thereafter captured employing Texture Exponent Software (Version 6.1.16.0) and considered indentation resistance or sample hardness.

##### In Vitro Drug Release

The in vitro drug release patterns of the EPHCD design formulations were evaluated in SUF over a duration of four weeks. Prior to the study, each EPHCD was secured to a 3D-printed T-shape plastic frame. Tinkercad^®^ software, a computer-aided design (CAD) tool, was utilized to design the T-shaped frame, which was then exported in .stl file format. Subsequently, Ultimaker Cura software (version 4.10.0, USA) was employed to slice the design prior to printing. The dimensions of the T-shaped frame included a diameter of 1 mm and arm lengths, as well as the vertical stem, each measuring 32 mm. For the 3D printing process, an Ultimaker2 printer (Ultimaker, Geldermalsen, The Netherlands) was employed, utilizing acrylonitrile butadiene styrene (ABS) filaments as the printing material.

All release assays were performed by placing the samples in SUF (pH 7; 37 °C; 15 mL) within an orbital shaker (25 rpm) to ensure consistent release dynamics. Eighty percent of the release medium was sampled every 24 h and promptly replaced with an equivalent volume of fresh medium to ensure unimpaired dissolution. Subsequently, all collected samples underwent filtration prior to analysis by UV spectroscopy at 280 nm (ε = 0.0064; R^2^ = 0.999) for quantification.

##### Constrained Statistical Optimization of the EPHCD

The optimization of the prepared EPHCD segment was conducted based on experimental design data. The optimized parameters utilized were “in range” for the independent variables, and a minimal E2 release and weight loss. The optimized EPHCD was thereafter subjected to in vitro assessment before inclusion in the MCDDS.

#### 2.2.2. In Vitro Characterization of the Optimal EPHCD

##### Determination of Chemical Integrity Using FTIR Spectroscopy Analysis

FTIR spectra were acquired for the optimal EPHCD, pure drug, and pristine polymer components utilizing an FTIR spectrophotometer (PerkinElmer Spectrum 100 FT-IR Spectrometer, Perkin Elmer, Waltham, MA, USA). For the FTIR study, the samples were subjected to 20 scans over 4000–650 cm^−1^ with all analyses undertaken at room temperature.

##### Morphological Analysis

Surface and cross-sectional imaging of the optimal EPHCD, both pre- and post-incubation in SUF for 8 weeks, was executed utilizing scanning electron microscopy (SEM) (ZEISS SEM, Carl Zeiss Microscopy Ltd., Cambridge, UK). For this, the sample specimens were affixed onto SEM sample holders and subjected to sputter coating with gold–palladium prior to analysis [8,23].

##### Water Retention Capacity

The optimized EPHCD and its corresponding DF formulations were evaluated for their hydration properties by employing a gravimetric approach. Briefly, all samples were weighed initially while dry (*W_i_*) using a digital balance. Afterwards, each sample was immersed in distilled water at room temperature (22 °C), for 24 h. Thereafter, the formulations were retrieved and blotted with filter paper to remove the surface moisture, prior to reweighing (*W_F_*). The quantification of ensuing hydration percentage was calculated as per Equation (3).

(3) $$Hydration\;\%=\frac{W_{F}-W_{i}}{W_{i}}\times 100$$

##### Mathematical Modeling of the Release Kinetics

In order to determine the mechanism involved in E2 release from the optimal EPHCD, the release data were fitted against different release kinetic models, as described by Govender et al. [27]. The release models utilized for this modelling included the zero-order, first-order, Higuchi, Korsmeyer–Peppas, Hixson–Crowell, and Peppas–Sahlin models.

#### 2.2.3. Assembly of the MCDDS

The MCDDS IUD was assembled by envelopment of the optimized EPHCD and an NLPM, prepared as previously published [21], onto a 3D-printed T-shaped plastic frame. The non-erodible plastic structure was utilized to facilitate the insertion and removal of the platform in vivo, while ensuring the positioning of the NLPM unit in the upper uterus cavity, with the EPHCD positioned at the lower uterine cavity toward the cervix and vagina. The MCDDS was thereafter characterized for its drug release kinetics, ex vivo permeation potential, and cytocompatibility.

##### Drug Release of MCDDS

The drug release pattern of MCDDS in SUF over a four-week period was investigated as aforementioned. Drug quantification was performed using a high-performance liquid chromatography (HPLC) technique, adapted from a previously reported protocol [28], for simultaneous detection of E2 and NETA. Briefly, the HPLC system comprised a Flexar Binary LC pump and a Flexar UV/VIS LC detector (PerkinElmer Inc., Waltham, MA, USA) paired with a Brownlee Analytical C18 column (5 µm particle size, 4.6 mm diameter, 150 mm length; PerkinElmer, USA) operating at ambient temperature (22 °C). Chromatographic separation was attained via isocratic elution with a mobile phase comprising 60:40 acetonitrile to water, delivered at a flow rate of 0.8 mL per min, and each 20 µL injection was analyzed over a total run time of 12 min. The retention times for E2 and NETA were 3.26 and 8.66 min, respectively, with both drugs detected at 240 nm. Standard solutions of E2 and NETA were prepared by dissolving the drugs in SUF and diluting to appropriate concentrations. The peak areas exhibited a + linear relationship, with E2 and NETA concentrations ranging from 1.56 to 50 µg/mL. Data acquisition and analysis were performed using Chromera software (Version 4, PerkinElmer, USA). The cumulative drug release (n = 3) was determined based on HPLC data and normalized to the total drug content in the MCDDS.

##### Ex Vivo Drug Permeation Studies

Drug permeation across porcine uterine tissue was determined using a Franz diffusion cell (FDC) apparatus (PermeGear Inc., Bethlehem, PA, USA) equipped with a 12 mL receptor compartment. The porcine uterine tissues were procured from the Wits Research Animal Facility (WARF, School of Health Sciences, University of the Witwatersrand, Johannesburg, South Africa) under an approved waiver (Waiver 16-11-2023-O). Briefly, the uterine tissue was positioned between the donor compartment containing 1 mL SUF (pH 7), and the receptor compartment containing PBS (pH 7.4; 37 °C) of the FDC apparatus, with the endometrial part facing the donor compartment. In the donor compartment, a piece of the EPHCD and NLPM, correlating to the MCDDS, was placed, covered, and assessed for drug permeation. Thereafter, 0.2 mL was sampled hourly from the receptor compartment over an 8 h period, with fresh PBS replaced in the receptor compartment to maintain sink conditions. The drug flux through the uterine membrane was thereafter determined at steady-state per unit area using Equation (4).

(4) $$J_{s}=\frac{Q_{r}}{A\;t}$$

where *J_s_* is the drug flux (mg cm^−2^ h^−1^), *Q_r_* is the drug amount in mg that crosses via the pig tissue into the receptor compartment, *A* (cm^2^) represents the effective cross-sectional area available for drug permeation (1.039 cm^2^), and *t* (h) is the time of drug exposure to the tissues.

##### Cytocompatibility Studies

The cytocompatibility over a 7-day period for the optimal EPHCD and its DF counterpart, as well as for the combination of the optimized EPHCD and NLPM segments, was assessed on NIH/3T3 cells (mouse fibroblast cells), as reported by Abdelgader et al. [21]. Briefly, NIH/3T3 cells were cultured in DMEM supplemented with 10% fetal bovine serum (FBS) and 1% penicillin/streptomycin and maintained in a humidified incubator at 37 °C with 5% CO_2_. The cell culture media were replenished every 2 days until the cells reached approximately 90% confluency. After sterilization, both the EPHCD and the DF formulation were subjected to UV light exposure for 5 min, followed by ethanol washing (70%) for 15 s and subsequent drying [6]. Sample leachates were extracted under sterile conditions after incubation of the drug-loaded and DF devices in SUF, retained in a shaker (25 rpm; 37 °C), for 5 days. Cytocompatibility assessments of the samples (n = 5), after filtering through a sterile 0.45 μm syringe filter and dilution with cell culture media (1:5), were thereafter conducted using an MTT assay.

For the analysis, the cells were plated in 96-well plates at a density of 2 × 10^4^ cells per well and subsequently incubated at a temperature of 37 °C, with relative humidity of 95%, and 5% CO_2_, for 24 h. The cell culture media was then substituted with the appropriately diluted extracted leachates, and the cellular cultures were maintained for further intervals of 1, 3, 5, and 7 days. The diluted extracted leachates were refreshed every 2 days. MTT solution was introduced to each individual well, at a designated time period, followed by solubilization buffer after 4 h of incubation. The absorbance was then measured at 570 nm against a 690 nm background. Absorbance was quantified spectrophotometrically at 570 nm, with a background correction at 690 nm. Additionally, the cells were treated with DMSO and cell culture media as positive and negative controls, respectively. Similarly, cell viability in the presence of extracts from EPHCD and NLPM collected over 5 and 28 days in SUF was assessed. The cytocompatibility of the MCDDS was thereafter determined over a 7-day period using the methodology described above.

#### 2.2.4. Statistical Analysis

This study utilized Design Expert^®^ software to construct the CCD matrix, aiming at elucidating the independent factors’ effect on the responses of the prepared EPHCD, along with optimization strategies. Mean values ± standard deviation (SD) were calculated using Microsoft Excel 2010 software, and statistical analyses were performed utilizing SPSS version 16.0 for Windows. Normality of data distribution was further examined employing the Shapiro test, with the significance between two experimental groups determined using two-sample *t*-tests. Analysis of variance (ANOVA) followed by Games–Howell and Tukey HSD post hoc tests was additionally applied for result comparisons among three and more groups, where *p* ≤ 0.05 indicated statistical significance.

## 3. Results and Discussion

### 3.1. Fabrication of EPHCD

**Figure 2:**
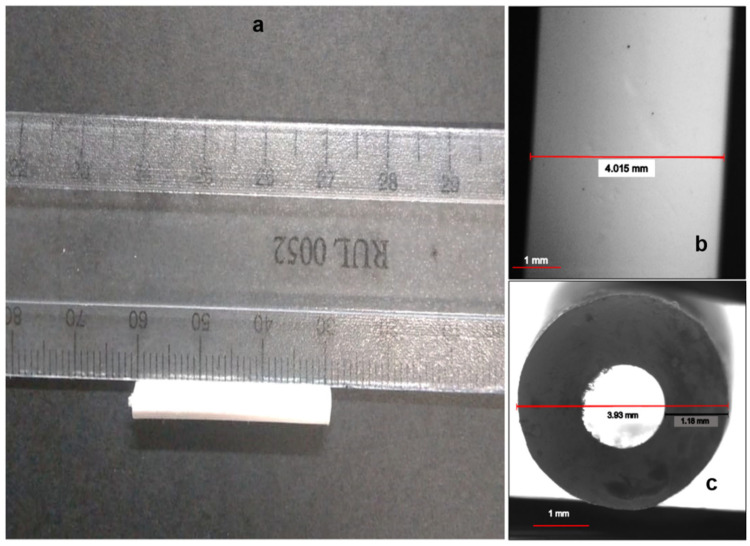
Photograph of the EPHCD: (**a**) front view in mm, (**b**) side view, and (**c**) cross-section view.

The formulation variables and processing methodologies adopted for the preparation of EPHCD were chosen based on preliminary investigations and an extensive review of relevant literature. Previous studies have highlighted PCL as an appropriate foundation for matrices intended for implantation into the female genitourinary system [1,29,30,31,32,33,34,35]. The range of polymer ratios investigated in this study, which was determined through preliminary evaluation, aimed to ease processing in terms of molding and demolding, achieving fracture-free device surfaces. Figure 2 depicts the successful fabrication of EPHCD, characterized by an average dimension of 30 mm length, 4 mm diameter, and a 1.5 mm inner diameter.

### 3.2. Evaluation of the Experimental Design

#### 3.2.1. Determination of the EPHCD Diameter and Uniformity of Drug Content

The evaluation of drug content uniformity provides crucial insights into the homogeneity of drug distribution through the different formulations and within individual devices. Elucidated in Table 2 are the mean diameter and drug content of the EPHCD formulations. The results demonstrate that E2 content closely conforms to the original feed percentage across the various formulations, exhibiting a minimal SD, less than the pharmacopoeial specification (15%) [36]. This observation indicates a consistent and even distribution of E2 contained by the polymer matrices. Additionally, the drug content analysis reveals a direct correlation between the amount of drug utilized in the device preparation and the resulting drug content. This could be due to the hydrophobic nature of the E2 and the properties of the polymers used in fabrication of EPHCD. Consequently, the outcomes pertaining to content uniformity and diameter analysis indicate that the employed preparation methodology and processing parameters adhere to standards indicative of reproducibility, rendering them suitable and acceptable for consistent manufacturing practices [23,37].

#### 3.2.2. EPHCD Degradation

Monitoring of the matrix erosion in SUF is vital in platforms such as EPHCD due to its potential impact on drug release. Results of the percentage weight loss analysis of the design formulations (EH1 to EH14), which correlated with matrix erosion, ranged from 1.66 ± 0.35 to 3.06 ± 0.38%, as shown in Figure S1 provided in the Supplementary Materials. Notably, formulations with a low drug load (5%), such as EH1 and EH3, showed a significant decrease in weight loss percentage with increasing EC content from 10% to 50%. Conversely, in formulations with a high drug load (10%), such as EH2 and EH4, an increment in EC content led to an observable increase in the percentage of weight loss. Additionally, at 10% EC, a shift from a 5% to 10% drug load resulted in a decrease in weight loss percentage, observed in formulations EH1 and EH2, while in contrast, at 50% EC, an increase in drug load (5% to 10%) increased the weight loss percentage, as seen in formulations EH3 and EH4. Interestingly, intermediate levels for both drug-load and polymer ratio resulted in the most significant reduction in weight loss.

**Figure 3:**
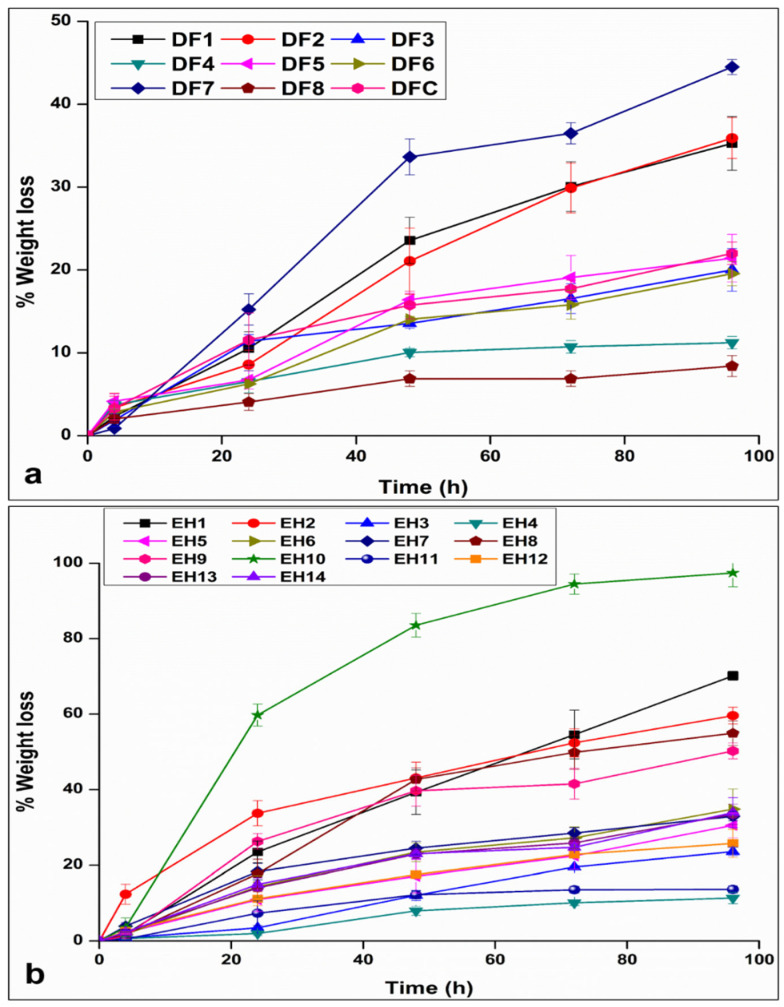
Weight loss % in high-pH media as a function of time of (**a**) the DF design devices and (**b**) the EPHCD design formulations. The data for DF1, DF2, DF3, DF4, and DFC were reproduced from Abdelgader et al. [21], © 2024 by the authors, Licensee MDPI, Basel, Switzerland.

Assisted alkaline degradation additionally offers a more physiologically relevant simulation compared to alternative methods like temperature-based acceleration [38]. The impact of E2 load and polymer ratio on the degradation EPHCD, as well as impact of polymer ratio on the degradation of the DF devices, were analyzed employing 5 M NaOH (Figure 3). The findings emphasized a decrease in weight loss with a corresponding increase in EC content, as notable when comparing DF7 and EH10 versus DF8 and EH4, respectively. In addition, a discernible pattern emerged when comparing the weight loss of formulations with 10% EC-to-PCL ratio. Specifically, EH1 and EH2 showed a significant increase in weight loss when compared to their DF equivalents DF1 and DF2 (in all cases, *p* = 0.000). However, when comparing EH4 (high EC content and 10% E2 load) to its DF formulation, no significant variations in weight loss were observed after 4 days (*p* = 0.985). These results therefore suggested that the EC content employed in the fabrication of the devices played a pivotal role in shaping the erosion behaviors of the EPHCD formulations. This could be attributed to EC’s known hydrophobic properties and structural rigidity [39,40,41], with an increase in EC content within the matrix corresponding to an escalation in hydrophobicity. Furthermore, the escalation of EC content enhanced the interfacial adhesion between the device components. This collective impact of heightened hydrophobicity and enhanced interfacial adhesion resulted in the formation of a protective barrier, impeding the ingress of water into the matrix, as well as growing a denser and even structural arrangement, minimizing the presence of micro-voids, and consequently sites for water penetration, thereby obstructing the onset and progression of the degradation mechanism.

#### 3.2.3. Textural Analysis

**Figure 4:**
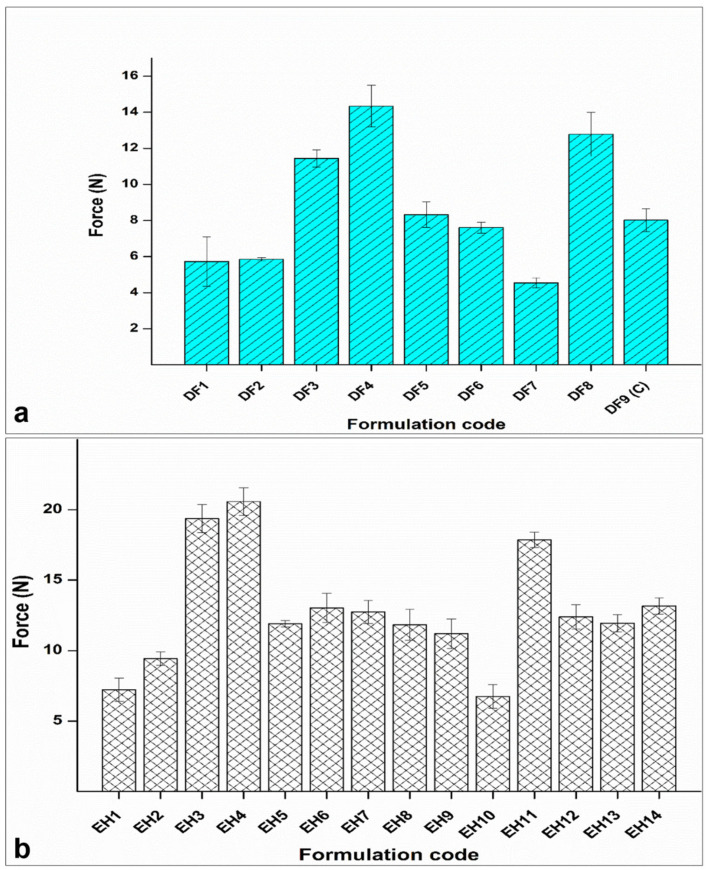
Analysis of indentation force of (**a**) the DF design devices and (**b**) the EPHCD design formulations. The data for DF1, DF2, DF3, DF4, and DF9 (C) were reproduced from Abdelgader et al. [21], © 2024 by the authors, Licensee MDPI, Basel, Switzerland.

The textural analysis results of DF devices displayed a significant effect of increasing EC levels on the penetration force required to indent the polymeric matrices (Figure 4a). This influence is manifest when comparing formulations DF4 and DF8, representing 50% and 58.28% EC-to-PCL ratio, respectively, with those containing low (10%, DF1 and DF2) as well as extra-low levels of EC (1.72%, DF7) (*p* = 0.011, 0.028, 0.016 between DF4 vs. DF1, DF2, and DF7, respectively and *p* = 0.025, 0.046, 0.027 between DF8 vs. DF1, DF2, and DF, respectively). Additionally, formulation DF9C, with a medium level of EC (30%), showed a slight, although not significant, increase in force compared to DF1 and DF2 (low level of EC; *p* = 0.416, 0.117 between DF9C vs. DF1 and DF2, respectively), a significant increase in force compared to DF7 (extra-low level of EC) (*p* = 0.027), and a significantly lower force compared to DF4 (high level of EC) (*p* = 0.023).

In EPHCD (Figure 4b), the resulting force values exhibited a significant increase with the escalation of ethyl cellulose (EC) content. Formulations EH11 (58.28%), EH4 (50%), and EH3 (50%), featuring high content of EC, displayed substantial differences in the indentation force compared to formulations with lower EC content (EH1, EH2, and EH10 of 10%, 10%, and 1.72% EC-to-PCL percentages, respectively), as well as those with a medium level of EC (center point formulations of 30% EC-to-PCL) (in all cases, *p* = 0.000). An increase in drug content further resulted in a minimal rise in force, evident in the comparisons between EH1 and EH3 versus EH2 and EH4 (5% and 10% E2 levels, respectively) (*p* = 0.119 between EH1 and EH2, *p* = 0.85 between EH3 and EH4). However, a significant impact on the force became apparent when transitioning from low and medium drug loads to the highest levels, accompanied by a decrease in EC from medium to the lowest level, as seen in the comparison between formulations EH9 and EH10 (*p* = 0.000). An interesting observation emerged when comparing formulations EH3, EH4, and EH11, representing 50% (EH3 and 4) and 58.28% (EH11) levels of EC. The change in drug load from the low level (EH3) to the medium level (EH11) and the high level (EH4) did not significantly affect the force. However, a significant influence on force was observed when transitioning from the medium level (EH11) to the high level (EH4) (*p* = 0.019). This result suggests a potential interaction effect between the two dependent variables as they fluctuate across upper and lower levels.

#### 3.2.4. In Vitro Drug Release

**Figure 5:**
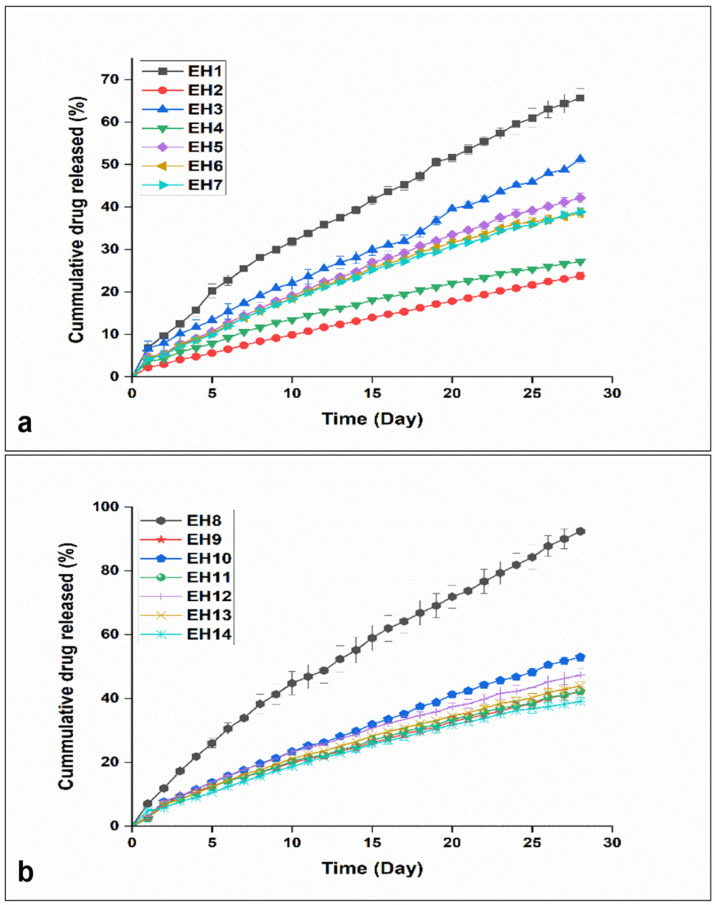
The release profiles of the EPHCD design formulations: (**a**) E1 to E7; (**b**) E8 to E14.

The cumulative percentages of drug release from EPHCD formulations (EH1 to EH14) over one month (Y4) displayed a notable variation from 23.78 ± 0.84 to 92.37 ± 2.91 (Figure 5). Particularly, formulations EH2 and EH4, characterized by high drug loads (10%) combined with low (10%) and high EC (50%) ratios, respectively, demonstrated relatively lower release percentages. Conversely, formulation EH8, with the lowest E2 load (3.96%) and a medium EC ratio (30%), exhibited the highest release percentage. Additionally, a discernible trend surfaced when scrutinizing formulations with a 5% drug load, showing an increased drug release from 51.29 ± 1.03 (EH3) to 65.65 ± 2.29 (EH1) with a corresponding shift in the EC percentage from 50 to 10%. It was also noted that formulations with medium levels of both drug load and polymer ratio displayed intermediate release percentages, as observed for EH5, EH6, EH7, EH12, EH13, and EH14. These results highlighted the significant impact of polymer composition and drug load on E2 release from EPHCD. It was further noted that, generally, EPHCD formulations featuring low E2 loads exhibited higher release rates compared to formulations with high E2 loading, consistent with prior findings [8]. The hydrophobic nature of E2 contributes to this trend, as increasing drug concentration enhances the hydrophobicity of the E2 polymer matrix. Consequently, this heightened hydrophobicity may impede the influx of release medium, thereby retarding drug release. Figure S2, provided in the Supplementary Materials, depicts the influence of both independent variables on cumulative E2 release percentage from the EPHCD design formulations.

In addition to the aforementioned, it is worth noting that the EPHCD was designed with the aim of sustaining the drug payload over an extended period of time (4 to 5 months); however, herein, the drug release was studied over 4 weeks, with the data obtained during this period used to predict the absolute cumulative drug release (100%) for the optimized formulation through drug release kinetics modelling.

#### 3.2.5. Data Analysis of the CCD and Statistical Optimization of the EPHCD

Table 3 summarizes the outcomes derived from the CCD, in conjunction with the experimental range. The data analysis of the experimental design and the optimization of the prepared devices has been provided in the Supplementary Materials (Section S.1, provided in the Supplementary Materials). The fitting of the multi-linear regression models to the acquired CCD results was also conducted, guided by the ANOVA results presented in Table S1 (Supplementary Materials). The findings demonstrate that the drug loading (X_1_) significantly influences both drug release (Y1, Y2, Y3, and Y4) and weight loss (Y5) percentages. Conversely, the EC-to-PCL percentage (X_2_) lacked statistically significant effects on drug release (%) throughout the test period, while the interaction between drug loading and EC-to-PCL percentage (X_1_X_2_) significantly influenced drug release (%) during the first 3 weeks. On other hand, both X_2_ and X_1_X_2_ do exert a significant influence on weight loss (%). Additionally, several quadratic terms displayed significant influences on the selected responses, with X_12_ impacting all responses, and X_22_, X_12_X_2_, and X_1_X_22_ affecting Y2 significantly. The plot of the measured and model-predicted values of the five responses, as depicted in Figure S3 (Supplementary Materials), illustrates a close alignment between the predicted values derived from the models and the actual experimental data, indicating the validity and robustness of the regression models. Also, the visual inspection of the normal probability plots, as depicted in Figure S4 (Supplementary Materials), reveals that the data points closely align with a linear pattern. This observation not only indicates the constructed equations’ viability to predict drug release and weight loss percentage but also implies their effectiveness in estimating the individual interactions between the responses and the independent variables, substantiating their adequacy.

The primary aim of employing the CCD-based optimization approach was to derive the optimal formulation parameters required for the preparation of the EPHCD. To achieve this goal, constraints were imposed on both the dependent and independent variables. Consequently, the CCD model generated an optimized formulation, corresponding to formulation EH2, indicating that the optimal formulation comprises a drug load of 10% and 10% EC-to-PCL ratio. This optimized formulation was projected to yield the optimum results for 1-, 2-, 3-, and 4-week percentage drug release as well as weight loss, achieving values of 9.7, 17, 25, 31.7, and 2.07%, respectively, as shown in Table S2 (Supplementary Materials). Furthermore, Figure S5a provides the contour plot of numerical optimization illustrating the selected optimum solution. Moreover, Figure S5b portrays the constructed desirability landscape, an optimal design space, achieved by superimposing contour plots and main effect plots derived by using the graphical optimization module of the implemented CCD.

### 3.3. Characterization of the Optimized EPHCD

#### 3.3.1. Fourier Transform Infrared (FTIR) Spectroscopy Analysis

**Figure 6:**
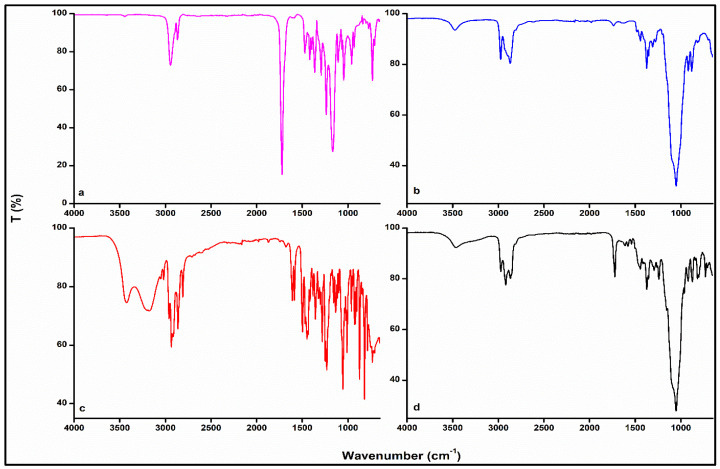
FTIR spectra of (**a**) PCL, (**b**) EC, (**c**) E2, and (**d**) the optimized EPHCD. The FTIR spectra of PCL and EC were reproduced from Abdelgader et al. [21], © 2024 by the authors, Licensee MDPI, Basel, Switzerland.

To ascertain the compatibility of the constituents within the fabricated optimized EPHCD formulation, FTIR spectra of the EPHCD samples were analyzed with respect to their individual components, encompassing E2, PCL, and EC (Figure 6). The E2 spectrum displayed broad bands at 3435 and 3194 cm^−1^, signifying OH stretching near the C-17 and C-3 positions. The broadness suggests hydrogen bonding with entrapped water. Additionally, peaks at 1609 and 1586 cm^−1^ indicate absorption by a mixture of tautomeric keto and enol forms. Furthermore, Figure 6 shows the characteristic peaks of PCL and EC, as detailed in our previous work [21]. The FTIR analysis of the EPHCD furthermore displayed the primary peaks associated with E2, although with reduced intensities. Concurrently, the characteristic peaks corresponding to the PCL and EC structures were observed. The consistent presence of identical characteristic peaks and the absence of discernible new peaks in the prepared device, compared to the pure drug and polymers, indicate a homogeneous blending of the device components. These findings support the absence of a strong chemical interaction and consequently affirm the compatibility of the device-forming polymers with the loaded drug.

#### 3.3.2. Morphological Analysis

**Figure 7:**
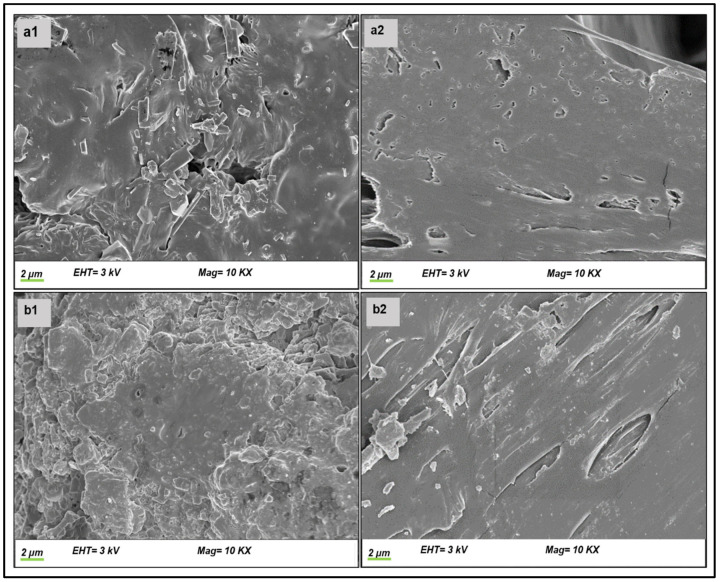
SEM analysis of surface and cross-sectional views, respectively, for (**a1**,**a2**) the EPHCD and (**b1**,**b2**) the 2-month degraded EPHCD.

SEM imaging was used to assess the surface morphological structures of the optimized EPHCD, as well as any modification of the surface characteristics attributed to the erosion over the 8-week period (Figure 7). SEM analysis of EPHCD unveiled minimal surface irregularities in both the surface and cross-section, with fewer cavities and pores, as well as evident drug crystals on the surface (Figure 7(a1,a2)). However, subsequent to incubation in SUF, the surface morphology (Figure 7(b1,b2)) exhibited cracked and rough surfaces, concomitant with notable cavities and the obliteration of pores, which could impede the penetration of dissolution medium into the EPHCD matrix.

#### 3.3.3. Water Retention Capacity

The hydration capacity investigations of the optimal DF and EPHCD devices were carried out over a 24 h period. The DF formulation exhibited a significantly higher hydration in comparison to the EPHCD (43.99% ± 1.44 vs. 26.23% ± 1.58, respectively; *p* = 0.000). This discrepancy may be due to the increased hydrophobicity of the matrix and a reduced porosity, which impeded water diffusion [42]. These findings provide additional insights into the observed low drug release in the optimized EPHCD formulation, highlighting the complex interplay between formulation characteristics and hydration behavior.

#### 3.3.4. Drug Release and Release Kinetics

The precise control of drug release stands as a paramount challenge in drug delivery, profoundly impacting treatment efficacy. Ensuring drug dosages align within a predefined range is imperative, with the lower threshold surpassing the therapeutic level and the upper limit remaining below toxic concentrations [43]. Prior research underscores that the kinetics of drug release from polymeric systems are governed by a multitude of factors, including polymer characteristics, environmental variables, and drug attributes. Furthermore, structural attributes of the system such as shape and size, as well as drug loading of polymeric matrices exert notable influences on the release kinetics [23,44]. Polymeric systems mediate drug release primarily through diffusion, erosion, and swelling mechanisms, while hydrophilic matrices facilitate drug release via water penetration, and consequently, the swelling and diffusion mechanisms are dominant. On the contrary, hydrophobic matrices predominantly rely on diffusion or erosion mechanisms, dictated by drug and excipient characteristics [44].

**Figure 8:**
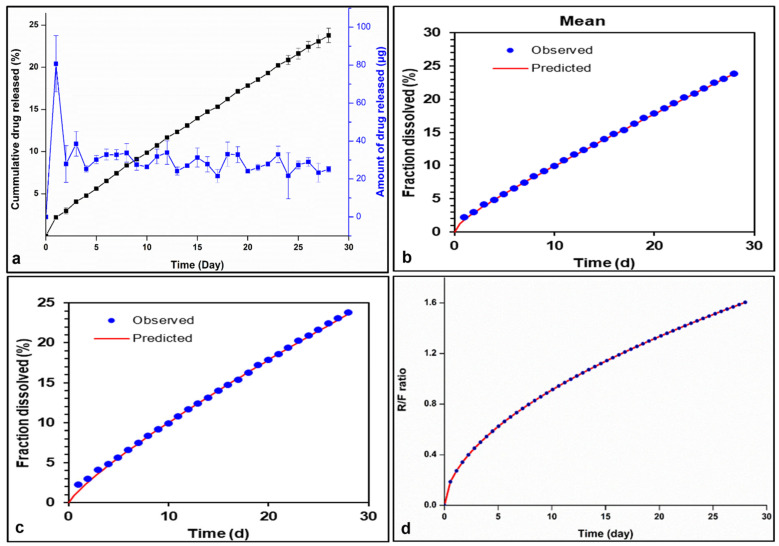
(**a**) Estradiol release profile from the optimized EPHCD formulation, EH2; fitting curve and modeling of E2 release from the optimal formulation. (**b**) Peppas–Sahlin and (**c**) Korsmeyer–Peppas models. (**d**) The ratio of relaxational to Fickian contributions as function of time.

The optimal formulation exhibited a desired sustained E2 release profile, marked by a cumulative release percentage of around 23% throughout the 4-week study. Notably, an initial burst release is observed on day 1, totaling 80.73 μg, followed by a rapid decrease in daily drug release during the first 3 days, transitioning into a much slower release rate over the subsequent 3 weeks, dropping below 30 μg after 21 days (Figure 8a). The observed burst release may stem from the release of surface-bound drug, which could be attributable to various chemical, physical, and processing parameters. Additionally, the emergence of surface cracks could facilitate surface erosion, further contributing to the burst release [45]. Analysis of the SEM images of the optimal EPHCD formulation revealed the presence of drug crystals on the EPHCD surface, along with low surface irregularities. The observed burst release on the first day could likely be attributed to these factors.

To delineate the mechanism of E2 release from the optimal EPHCD, the release kinetics were assessed by fitting the experimental data to various release kinetic models, including zero-order, first-order, Higuchi, Korsmeyer–Peppas, Hixson–Crowell, and Peppas–Sahlin models. Table 4 displays the goodness-of-fit outcomes, comprising R^2^, adjusted R^2^, RMSE, AIC, and MSC values, applied to the mean release profiles. Parameters derived from the nonlinear regression of the dataset fit to the models are also presented. Amongst the mathematical models applied to describe the drug release pattern of the optimal EPHCD, the Peppas–Sahlin model emerged as the most fitting, followed by the Korsmeyer–Peppas model, as indicated by R^2^ and adjusted R^2^ values exceeding 0.999, as well as AIC values below 8.41. Moreover, the models exhibited MSC values higher than 6.76, further highlighting their suitability for describing the release kinetics of the E2. Figure 8b,c illustrate the appropriateness of the best-fit models, Peppas–Sahlin and Korsmeyer–Peppas models, respectively, for accurately describing drug release profiles. In each of the figures, the time is represented on the horizontal axis, while the vertical axis represents the cumulative percentage of drug released over time. The chosen best-fit models closely align the predicted values with the actual experimental data, indicating their suitability for describing the drug release profiles.

In exploring the relation between the Peppas–Sahlin model and the dominant release mechanism, associations have been proposed between its parameters and the Korsmeyer–Peppas parameters. The release exponent “n” of the Korsmeyer–Peppas model for drug release from cylindrical polymeric delivery systems serves as a marker for the release mechanism [36,46], while the equality of the “n” value in the Korsmeyer–Peppas model and the “m” value in the Peppas–Sahlin model is anticipated when the relaxation mechanism is insignificant [45]. Thus, the dissimilarity between the n exponent (0.835) and the “m” exponent (0.55) highlights the co-dependence of diffusion and Case II relaxation in governing E2 release. Additionally, the attained release exponent “n” from the Korsmeyer–Peppas model was 0.835, corroborating that the mechanism of E2 release exhibits anomalous behavior or non-Fickian transport. This suggests that the release kinetics from EPHCD entails a complex process of diffusion, erosion and relaxation. Furthermore, the high kKP value from the Korsmeyer–Peppas model (1.464) suggested the presence of an initial burst release, with approximately 80 µg of E2 released after 24 h.

The ratio of relaxational to Fickian contribution, denoted as R/F, can be calculated using the following formula (Equation (5)):

(5) $$\frac{Relaxation\;contribution}{Fickian\;contribution}=\frac{k_{2}}{k_{1}}\;t^{m}$$

An R/F ratio of 1 denotes an equilibrium between the relaxation and diffusion processes within the release mechanism. Ratio values below 1 indicate that the diffusion plays the major role, whereas a ratio exceeding 1 signifies that the relaxation mechanism takes precedence [45]. The calculation of the R/F ratio was based on the parameters provided in Table 4, and the temporal variation of the R/F ratio is graphically represented in Figure 8d. The low R/F values initially indicate the prevalent influence of Fickian diffusion on drug release from the segment. The gradual increase in R/F ratio values over time signifies a growing role of relaxational contribution. Beyond day 12, when the R/F ratio surpasses 1, this marks the point at which the relaxation mechanism becomes the primary contributor.

### 3.4. Evaluation of the Combined MCDDS

**Figure 9:**
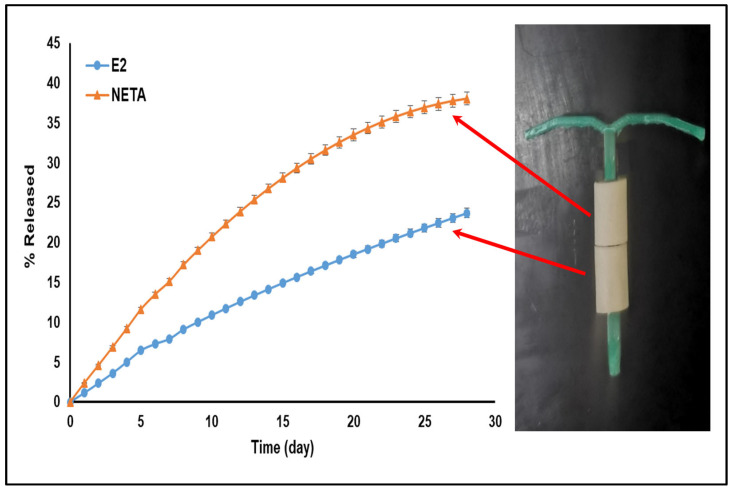
Assembly of the MCDDS and release profiles of E2 and NETA using HPLC analysis.

Figure 9 illustrates the assembled MCDDS, showcasing the arrangement with the optimal NLPM, positioned at the top, and EPHCD in the lower segment of the T-shaped plastic frame of the IUD. The results, as seen in Figure 9, revealed that the release of both drugs constituting the MCDDS was sustained over the four-week period, with the release profile data from MCDDS (23.67 ± 0.60% and 38.05 ± 0.82% for E2 and NETA, respectively) comparable to the release from each unit tested independently (23.78 ± 0.84% and 38.0 ± 1.60% for E2 and NETA, respectively). Moreover, extrapolating the release profiles of NETA and E2 using the Korsmeyer–Peppas equation, along with the obtained model parameters, reveals the platform’s capability to sustain the release of NETA for up to 15 weeks and E2 for up to 25 weeks (Figures S6a and S6b, respectively). A representative HPLC chromatogram for E2 and NETA has further been provided in the Supplementary Data (Figure S7a), as well as the standard curves generated through linear regression analysis (Figure S7b,c).

### 3.5. Ex Vivo Permeation

**Figure 10:**
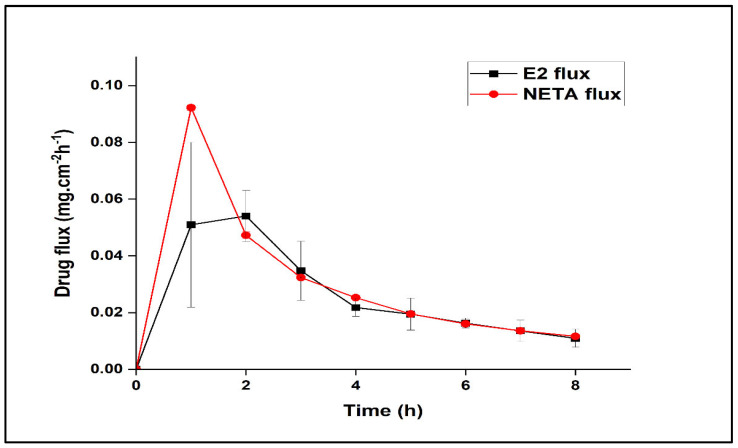
E2 and NETA flux through the uterine membrane as function of time.

The transport of a drug molecule across a biological membrane is significantly influenced by several crucial parameters. These include the molecules’ size, charge, lipophilicity, and hydrogen-bonding capacity. Physicochemical and structural attributes, such as molecular size, charge distribution, lipophilicity, and hydrogen-bonding capacity, play pivotal roles in determining the diffusional behavior of molecules across biological membranes [47]. As the accumulation of E2 and NETA in genitourinary tissues is crucial for exerting pharmacological effects and controlling GSM in a safe manner, their adequate accumulation was assessed by determining the flux of E2 and NETA released from the EPHCD and NLPM, respectively, through the uterine membrane. The flux of both hormones across porcine uterine tissue over 8 h is depicted in Figure 10. The results indicate an initial escalation in the rate of E2 flux up to (0.054 ± 0.009 mg cm^−2^ h^−1^) during the first 2 h and NETA flux up to (0.092± 0.001 mg cm^−2^ h^−1^) during the first hour, followed by a subsequent decrease in drug flux. This indicates that the mechanism of drug transport through the porcine uterine membrane is hindered by binding and accumulation of E2 and NETA onto the membrane after 2 and 1 h, respectively. Therefore, it can be suggested that most of the drug was adsorbed onto the surface of the tissue, enabling localized effects in the uterus and potential washing out through uterine fluid to translocate and exert therapeutic effects in the vaginal tissue. However, the possibility of systemic absorption cannot be excluded. In the event of systemic absorption, this may expand the therapeutic applicability beyond GSM. Nonetheless, further studies are warranted to assess the extent of systemic exposure, as well as to elucidate any potential systemic therapeutic benefits and associated adverse effects.

### 3.6. Cytocompatibility Studies

**Figure 11:**
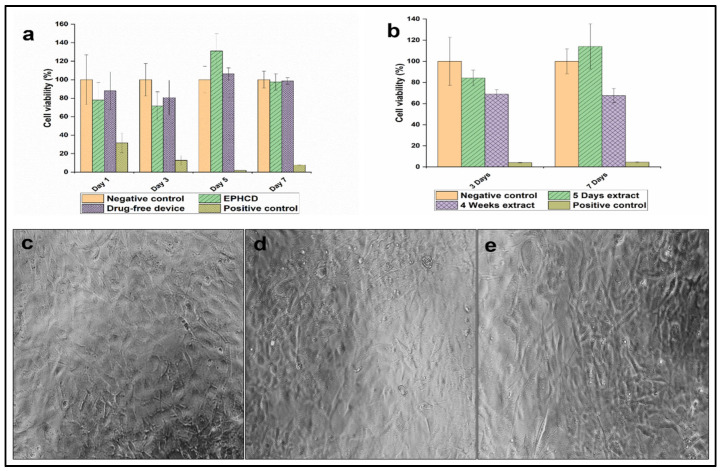
Cellular viability of NIH/3T3 by MTT assay in the presence of (**a**) the optimal EPHCD and DF formulations, (**b**) the mixture (1:1) of 5-days and 4-week extracts of the EPHCD and NLPM, in addition to micrographs of NIH/3T3 cells after 7-day treatment at 20× for (**c**) the negative control in cell culture media, (**d**) the DF device extract, and (**e**) EPHCD extract (scale: 1 cm = 100 µm). Data and image of negative and positive control were reproduced from Abdelgader et al. [21], © 2024 by the authors, Licensee MDPI, Basel, Switzerland.

Cytotoxicity studies stand as pivotal and extensively employed protocols for evaluating cyto-compatibility, recognized for their rapidity, sensitivity, and methodological simplicity [48]. The cytocompatibility of the optimal EPHCD and its respective DF counterpart was evaluated by assessing the cellular viability of NIH/3T3 cells. The negative control, as reported in our previous study [21], comprised the cell culture medium with the resultant cell viability normalized to 100% for comparison with the test samples. Figure 11a depicts the results of an in vitro cell study using the MTT assay over a 7-day period, with both EPHCD and DF devices demonstrating cell viability exceeding 70%, indicating the non-toxic and cytocompatible nature of the prepared formulations in this investigation [48,49,50]. Evaluation of the cells 1 and 7 days post-treatment showed that both treatments demonstrated a non-significant effect over the cell viability in comparison to the viability of the negative control, [*p* = 0.095, *p* = 0.561 (Day 1) and *p* = 0.925, *p* = 0.968 (Day 7) for EPHCD and DF device, respectively]. On day 3, both devices demonstrated significant differences in comparison to the negative control (*p* = 0.001, *p* = 0.043, for EPHCD and DF device, respectively), while on day 5, the EPHCD-treated cells exhibited more significant cellular viability compared to the DF device and to the negative control [*p* = 0.009, *p* = 0.003], respectively. Figure 11c–e display photomicrographs captured on day 7 after treatment of the negative control, EPHCD extract, and DF device extract, respectively, further affirming the cytocompatibility of the optimal EPHCD formulation.

Figure 11b illustrates the cellular viability of NIH/3T3 cells following treatment with extracts obtained from the MCDDS for 5 days and 4 weeks. Assessment at 3 and 7 days post-treatment revealed that the 5-day mixture extract showed cell viability above 70%, indicative of the non-toxic and cytocompatible nature of the prepared formulations. Conversely, the 4-week mixture extract exhibited cellular viability of 68.9 ± 4.27% and 67.53 ± 6.76% post-incubation for 3 and 7 days with NIH/3T3 cells, respectively. This cellular viability, below the 70% threshold, could be attributed to high cumulative drug concentrations following the 4-week extraction period, which may not accurately mimic the in vivo conditions of the genitourinary tract’s self-washing mechanism.

## 4. Conclusions

This study has presented the development and analysis of an E2-loaded continuous- and sustained-release polymeric hollow cylindrical device as an estrogenic intervention to address GSM. Evaluation of the prepared EPHCD system underscored the crucial roles of drug load and the ratio of the polymers constituting the prepared matrix in modulating drug release kinetics and device integrity. The CCD analysis further revealed notable differences in device properties arising from variations in the levels of drug quantity and the EC-to-PCL percentage. These properties were evaluated in terms of drug release and weight loss, with ANOVA and response surface plots affirming the significant impact of factor levels on the measured parameters. The close alignment between experimental and fitted values underscored the precision and reliability of the used statistical design. The optimization process used targeted both percentage drug release and weight loss, with particular emphasis on the latter due to its considerable impact on matrix integrity and drug release kinetics. The release kinetics of E2 from the optimal EPHCD adhered to Peppas–Sahlin and Korsmeyer–Peppas kinetics, indicative of an intricate interplay between diffusion and polymer relaxation. The optimized E2-loaded device was further combined with the NLPM to form the MCDDS IUD, which showed the potential to sustain its E2 and NETA cargo for about 25 and 15 weeks, respectively. In addition, permeation study findings suggested localized genitourinary tract therapeutic effects due to the in situ accumulation of both drugs in the uterine tissues rather than systemic absorption. Moreover, cytocompatibility studies using leachates of the MCDDS IUD noted that at low concentration of the drug, which will be maintained in vivo through natural self-washing processes, the device is appropriate for the long-term management of GSM. Overall, the outcomes of this study position the established platform as a potential candidate for the sustained release of E2 and NETA into the uterine cavity, a feature that can be finely modulated by choice of drug load and polymer ratio. This functionality presents a viable platform for addressing GSM, with potential implications for advanced biomedical applications such as contraception in women of child-bearing age. Additional research, which is currently being conducted, includes expanding the investigation across two anatomical distinct body compartments, through tailoring a single multi-unit platform for implantation of the E2- and NETA-loaded unit into the vaginal and uterus cavity, respectively, while further research which can be undertaken includes the analysis of the cytocompatibility of the prepared MCDDS platform using additional cell lines stemming from the genitourinary tract, as well as in vivo studies in a GSM animal model.

## Figures and Tables

**Table 1:** Experimental design domain of the CCD and compositions of the DF comparator units.

EPHCD			Point Type	Drug-Free (DF) Device	
F Code *	E2	EC-to-PCL Ratio		F Code *	EC-to-PCL Ratio
EH1	5% (−1)	10% (−1)	Factorial	DF1	10% (−1)
EH2	10% (1)	10% (−1)	Factorial	DF2	10% (−1)
EH3	5% (−1)	50% (1)	Factorial	DF3	50% (1)
EH4	10% (1)	50% (1)	Factorial	DF4	50% (1)
EH5	7.5% (0)	30% (0)	Center		
EH6	7.5% (0)	30% (0)	Center		
EH7	7.5% (0)	30% (0)	Center		
EH8	3.96% (−1.414)	30% (0)	Axial	DF5	30% (0)
EH9	11.04% (1.414)	30% (0)	Axial	DF6	30% (0)
EH10	7.5% (0)	1.72% (−1.414)	Axial	DF7	1.72% (−1.414)
EH11	7.5% (0)	58.28% (1.414)	Axial	DF8	58.28% (1.414)
EH12	7.5% (0)	30% (0)	Center	DF9 (C) **	30% (0)
EH13	7.5% (0)	30% (0)	Center		
EH14	7.5% (0)	30% (0)	Center		

* Formulation code. ** Center point formulation.

**Table 2:** Diameter and drug content of the prepared EPHCD design formulations (n = 3).

Formulation Code	Diameter (mm)	Original Loading%	Drug Content (% ± SD)	Loading Efficiency (% ± SD)
EH1	3.76 ± 0.08	5	4.56 ± 0.34	91.24 ± 6.70
EH2	4.07 ± 0.01	10	9.17 ± 0.50	91.69 ± 5.04
EH3	4.07 ± 0.01	5	4.46 ± 0.46	89.15 ± 9.10
EH4	4.13 ± 0.04	10	9.97 ± 0.02	99.73 ± 0.21
EH5	4.09 ± 0.02	7.5	7.26 ± 0.34	96.79 ± 4.55
EH6	4.10 ± 0.01	7.5	7.01 ± 0.17	93.41 ± 2.20
EH7	4.07 ± 0.01	7.5	7.14 ± 0.34	95.23 ± 4.53
EH8	3.85 ± 0.03	3.96	3.89 ± 0.09	98.33 ± 2.22
EH9	4.04 ± 0.05	11.04	10.79 ± 0.24	97.77 ± 2.13
EH10	3.97 ± 0.03	7.5	7.29 ± 0.12	97.18 ± 1.60
EH11	4.07 ± 0.05	7.5	7.34 ± 0.10	97.91 ± 1.37
EH12	4.06 ± 0.04	7.5	7.16 ± 0.12	95.39 ± 1.62
EH13	4.05 ± 0.05	7.5	7.31 ± 0.09	97.49 ± 1.24
EH14	4.10 ± 0.01	7.5	7.09 ± 0.22	94.49 ± 2.92

**Table 3:** CCD experimental design in vitro drug release analysis results (n = 3, ±SD).

Formulation Code	1-Week Drug Release (%)	2-Week Drug Release (%)	3-Week Drug Release (%)	4-Week Drug Release (%)	8-Week Weight Loss
EH1	25.49 ± 0.95	39.28 ± 0.88	53.53 ± 1.11	65.65 ± 2.29	2.48 ± 0.44
EH2	7.43 ± 0.03	13.09 ± 0.17	18.54 ± 0.16	23.78 ± 0.84	2.09 ± 0.21
EH3	17.34 ± 1.72	28.07 ± 1.45	40.39 ± 0.81	51.29 ± 1.03	1.82 ± 0.44
EH4	10.58 ± 0.48	16.91 ± 0.41	22.63 ± 0.38	27.17 ± 0.27	2.38 ± 0.52
EH5	14.39 ± 0.26	24.72 ± 0.67	34.52 ± 1.02	42.10 ± 1.12	2.73 ± 0.41
EH6	13.84 ± 0.20	23.68 ± 0.50	32.53 ± 0.76	38.55 ± 1.04	2.95 ± 0.08
EH7	13.93 ± 0.46	23.35 ± 0.16	31.66 ± 0.27	38.89 ± 0.87	3.06 ± 0.38
EH8	33.89 ± 2.54	55.18 ± 4.04	73.66 ± 3.89	92.37 ± 2.91	1.80 ± 0.14
EH9	15.49 ± 0.55	24.58 ± 0.90	33.82 ± 1.26	42.07 ± 1.61	2.76 ± 0.41
EH10	17.56 ± 0.63	29.81 ± 0.23	42.36 ± 0.82	53.03 ± 1.33	2.74 ± 0.38
EH11	15.51 ± 0.58	25.03 ± 1.02	34.39 ± 1.51	42.26 ± 1.76	1.66 ± 0.35
EH12	17.80 ± 0.42	28.82 ± 0.64	38.35 ± 1.38	47.33 ± 2.00	2.85 ± 0.18
EH13	16.22 ± 0.08	26.49 ± 0.25	35.55 ± 0.84	43.91 ± 1.38	2.75 ± 0.11
EH14	14.06 ± 0.49	23.95 ± 0.78	32.71 ± 1.34	39.09 ± 1.30	2.76 ± 0.07

**Table 4:** Statistical assessment and parameters for the best-fitting kinetic models to release data for optimal EPHCD.

Model	R^2^	Adj-R^2^	RMSE	AIC	MSC	Parameter	
Zero-order	0.9816	0.9816	0.892	87.90	3.924	k_0_	0.892
First-order	0.9926	0.9926	0.567	62.55	4.829	k_1_	0.010
Higuchi	0.8938	0.8938	2.144	136.99	2.171	kH	3.855
Korsmeyer–Peppas	0.9990	0.9990	0.212	8.41	6.763	kKP	1.464
						n	0.835
Hixson–Crowell	0.9898	0.9898	0.665	71.46	4.511	kHC	0.003
Peppas–Sahlin	0.9997	0.9997	0.111	−27.15	8.033	k_1_	1.466
						k_2_	0.376
						m	0.55

## Data Availability

The original contributions presented in this study are included in the article. Further inquiries can be directed to the corresponding author.

## Supplementary Materials

The following supporting information can be downloaded at: www.mdpi.com/article/10.3390/polym17050665/s1, Figure S1: 3D plot portraying the influence of E2 load (%) and EC-to-PCL (%) on the weight loss percentage of the EPHCD; Figure S2: 3D Surface plots depicting the influence of E2 load (%) and EC-to-PCL (%) on the cumulative drug release at (a) week 1, (b) week 2, (c) week 3, and (d) week 4; S.1: Data analysis of the experimental design; Table S1: Analysis of variance (ANOVA) for different responses of EPHCD formulations; Figure S3: Plot of the measured and model-predicted values of the response (a) Y1, (b) Y2, (Y3), (Y4), and (Y5) of EPHCDs; Figure S4: The normal probability plot of the residuals for (a) Y1, (b) Y2, (c), Y3 (d) Y4, and (e) Y5 of EPHCDs; S.2: Constrained statistical Optimization of the EPHCD; Table S2: The selected optimal configuration from numerical optimization; Figure S5: Contour plot of numerical optimization (a) and overlay plot of graphical optimization (b); Figure S6: The expected release for (a) NETA and (b) E2 from MCDDS, using the Korsmeyer-Peppas model; Figure S7: (a) HPLC chromatogram of E2 and NETA, (b) standard curve of E2, and (c) standard curve of NETA.

## Abbreviations

The following abbreviations are used in this manuscript:

ABS	Acrylonitrile butadiene styrene
AIC	Akaike information criterion
CCD	Central composite design
CAD	Computer-aided design
DoE	Design of experiments
DF	Drug-free formulation
E2	Estradiol hemihydrate
EPHCD	Estradiol-loaded polymer-based hollow cylindrical delivery system
EC	Ethyl cellulose
FDC	Franz diffusion cell
GSM	Genitourinary syndrome of menopause
IDDS	Implantable drug delivery systems
IUD	Intrauterine device
MSC	Model selection criterion
MCDDS	Multi component drug delivery system
NETA	Norethindrone acetate
NLPM	Norethindrone acetate-loaded hollow cylindrical polymeric matrix
PBS	Phosphate-buffered saline
PCL	Polycaprolactone
PRESS	Predicted residual sum of squares
SEM	Scanning electron microscopy
SUF	Simulated uterine fluid
SD	Standard deviation
