# Supplementary material for: A Novel Intrauterine Device for the Extended Tissue-Specific Release of Estradiol and Norethindrone to Treat the Genitourinary Syndrome of Menopause
Source: Polymers (Basel). 2025 Feb 28;17(5):665. doi: 10.3390/polym17050665 (PMC11902457; doi:10.3390/polym17050665)
Supplement: Supplementary file 1 [file polymers-17-00665-s001.zip › polymers-3477641-supplementary.pdf]

polymers

MDPI

Supplementary Material

A Novel Intrauterine Device for the Extended Tissue-Specific Release of Estradiol and Norethindrone to Treat the Genitourinary Syndrome of Menopause

Ahmed Abdelgader, Mershen Govender, Pradeep Kumar and Yahya E. Choonara *

Wits Advanced Drug Delivery Platform Research Unit, Department of Pharmacy and Pharmacology, School of Therapeutic Sciences, Faculty of Health Sciences, University of the Witwatersrand, Johannesburg, 7 York Road, Parktown, Johannesburg 2193, South Africa; pradeep.kumar@wits.ac.za (P.K.)
* Correspondence: yahya.choonara@wits.ac.za; Tel.: +27-11-717-2052; Fax: +27-11-642-4355

Academic Editors: Panagiotis Barmpalexis and Afroditi Kapourani

Received: 29 January 2025
Revised: 26 February 2025
Accepted: 27 February 2025
Published: 28 February 2025

Citation: Abdelgader, A.; Govender, M.; Kumar, P.; Choonara, Y.E. A Novel Intrauterine Device for the Extended Tissue-Specific Release of Estradiol and Norethindrone to Treat the Genitourinary Syndrome of Menopause. *Polymers* **2025**, *17*, 665. https://doi.org/10.3390/polym17050665

Copyright: © 2025 by the authors. Submitted for possible open access publication under the terms and conditions of the Creative Commons Attribution (CC BY) license (https://creativecommons.org/licenses/by/4.0/).

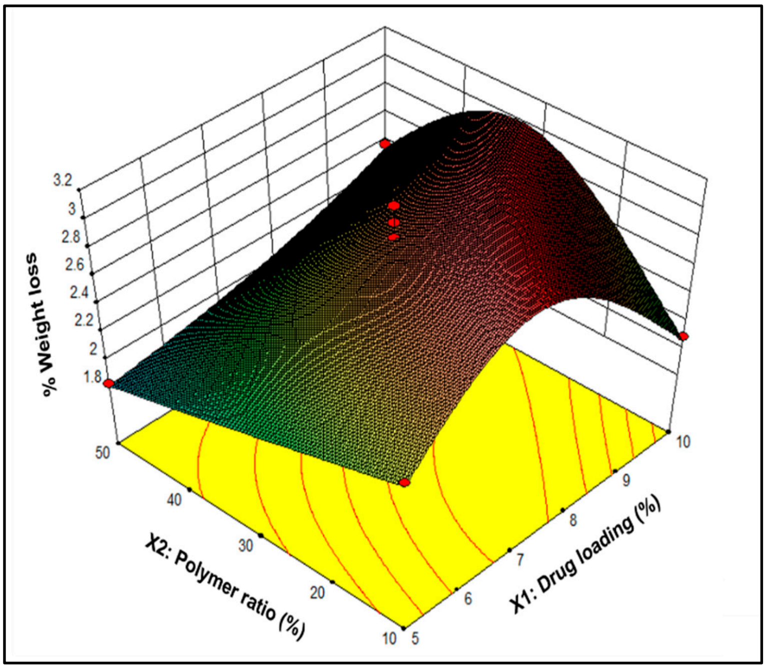

Figure S1: 3D plot portraying the influence of E2 load (%) and EC-to-PCL (%) on the weight loss percentage of the EPHCD.

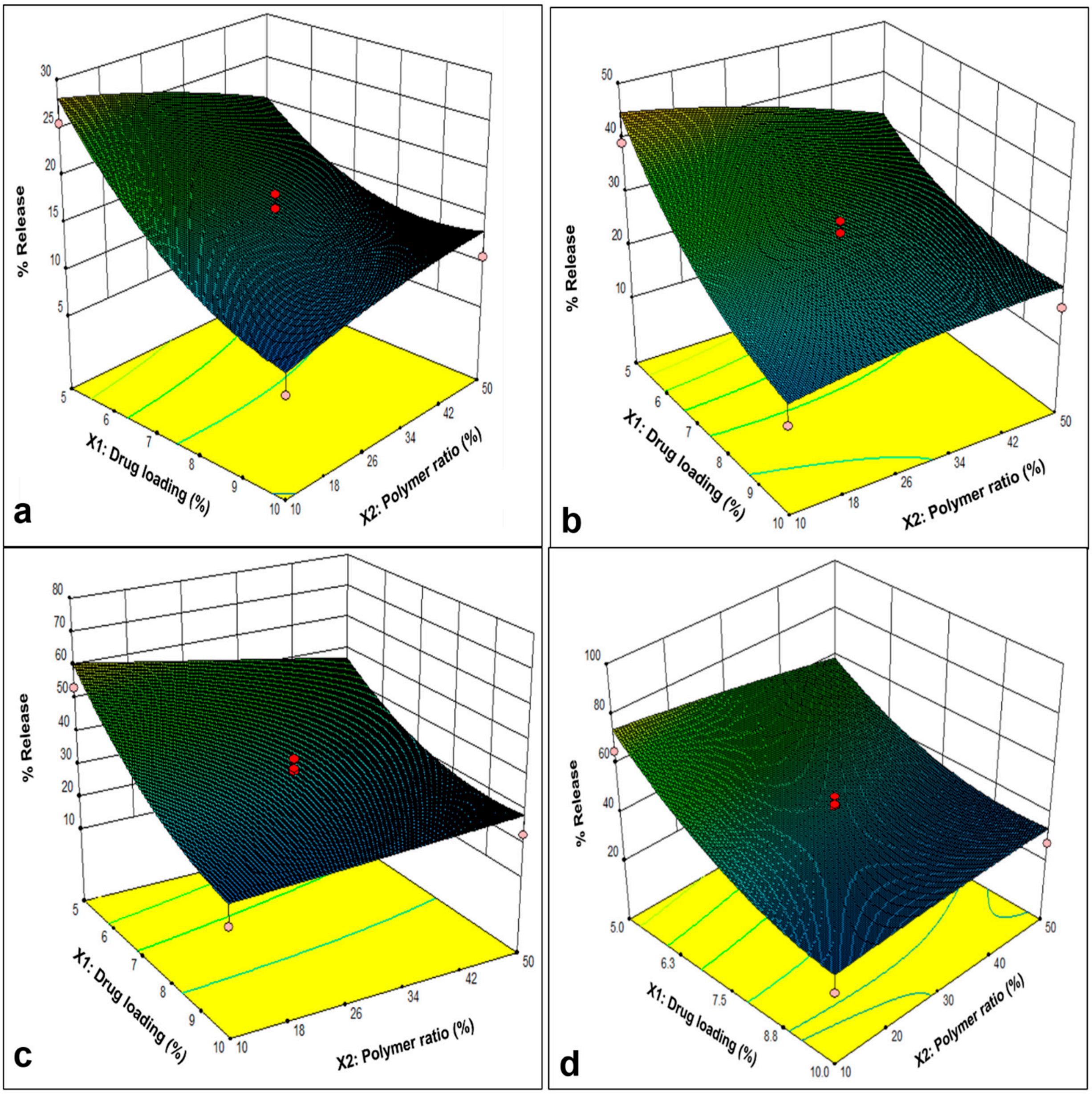

Figure S2. 3D Surface plots depicting the influence of E2 load (%) and EC-to-PCL (%) on the cumulative drug release at (a) week 1, (b) week 2, (c) week 3, and (d) week 4.

S.1. Data analysis of the experimental design

Table 3 presents a summary of the outcomes derived from the CCD, in conjunction with the experimental range. The findings revealed that the 1, 2, 3, and 4-week percentage released of drug (Y1, Y2, Y3, and Y4) ranged from 7.43% to 33.89%, 13.09% to 55.18%, 18.54% to 73.66%, and 23.78% to 92.36%, respectively, while the 8-week weight loss percentage (Y5) ranged from 1.66% to 3.06%. This suggests that the chosen upper and lower levels for the factors were effective in generating a broad experimental domain. Both of the independent variables were encoded within the range of -1 to 1. To meet the validity requirements of ANOVA, the experimental data pertaining to Y2, Y4, and Y5 responses underwent transformations, specifically, the square root transformation for Y2 and Y4, while the inverse transformation for Y5. The fitting of the multi-linear regression models to the acquired CCD results was conducted, guided by the ANOVA results

presented in Table S1. The remarkable low probability values (p-value < 0.0001 for Y1, Y3, Y4, Y5, and < 0.0003 for Y2) underscore the robust significance of the models developed for both responses, validated at a 95% confidence interval (where p-values below 0.05 denote significance). The absence of a significant lack-of-fit further emphasizes the high predictive capability of the model. Specifically, the Lack of Fit p-values of 0.2569, 0.1064, 0.0895, 0.1506, and 0.7573 for Y1, Y2, Y3, Y4, and Y5, respectively, strongly suggest their minimal influence in comparison to pure error. The findings demonstrate that the drug loading (X_1) significantly influences both drug release (Y1, Y2, Y3, and Y4) and weight loss (Y5) percentages. Conversely, the EC-to-PCL percentage (X_2) lack statistically significant effects on drug release (%) throughout the 4 weeks, while the interaction term between drug loading and EC-to-PCL percentage (X_1X_2) significantly influence the drug release (%) during the first 3 weeks and lack the influence in the week 4. On other hand, both X_2 and X_1X_2 do exert a significant influence on weight loss (%). Additionally, several quadratic terms displayed significant influences on the selected responses, with X_1^2 impacting all responses, and X_2^2, $X_1^2X_2$, and $X_1X_2^2$ affecting Y2 significantly.

An array of fit statistics, including R^2, adjusted R^2, predicted R^2, adequate precision, Coefficient of Variation (CV), and Predicted Residual Sum of Squares (PRESS), are determined and analyzed. The predicted and experimental responses demonstrated a good fit, with R^2 measuring more than 0.94. Furthermore, all responses exhibited adequate precision values exceeding 4, and the predicted R^2 values closely aligned with the adjusted R^2 values, with differences of less than 0.2. Additionally, coefficient of variations were less than 10, and model selection prioritized minimizing PRESS values. Moreover, the analysis of CCD results led to the derivation of the following second-order polynomial equations, expressed in terms of coded factors:

$$Y1 = +15.04 - 6.36\,X_1 - 0.99\,X_2 + 2.83\,X_1X_2 + 3.47\,X_1^2 - 0.6\,X_2^2 \quad \text{(S1)}$$

$$Sqrt\,(Y2) = +5.01 - 0.92\,X_1 - 0.14\,X_2 + 0.37\,X_1X_2 + 0.37\,X_1^2 - 0.11\,X_2^2 \quad \text{(S2)}$$

$$Y3 = +33.61 - 13.64\,X_1 - 2.54\,X_2 + 4.31\,X_1X_2 + 6.76\,X_1^2 \quad \text{(S3)}$$

$$Sqrt\,(Y4) = +6.41 - 1.2\,X_1 - 0.21\,X_2 + 0.32\,X_1X_2 + 0.52\,X_1^2 \quad \text{(S4)}$$

$$1/Y5 = +0.35 - 0.069\,X_1 + 0.084\,X_2 - 0.051\,X_1X_2 + 0.052\,X_1^2 + 0.064\,X_2^2 - 0.062\,X_1^2\,X_2 + 0.055\,X_1X_2^2 \quad \text{(S5)}$$

The polynomial equations were employed to draw conclusions based on the magnitude and mathematical sign of the coefficients. In Equations S1, S2, S3, and S4, both

the main effect terms X_1 and X_2 displayed a negative effect on the responses (Y1, Sqrt (Y2), Y3, and Sqrt(Y4)), with the drug load exerting a significant dominant influence. Conversely, the interaction terms X_1X_2 and the quadratic term X_1^2 had positive impact on the responses, with the X_1^2 term demonstrating a significant dominant effect. Furthermore, the quadratic term X_2^2 displayed slight non-significant positive effects on the Y1 and Sqrt (Y2). In Equation S5, It appears that inverse of weight loss percentage has a negative relationship with the X_1, X_1X_2, and $X_1^2X_2$ as main effect, interaction and quadratic terms, respectively. On the contrary, the X_2, X_{12}, X_{22}, and X_1X_{22} exhibited a significant positive impact on the inverse of the weight loss percentage.

Table S1. Analysis of variance (ANOVA) for different responses of EPHCD formulations.

	**Y1**	**Y2**	**Y3**	**Y4**	**Y5**
**Source**	P-value	P-value	P-value	P-value	P-value
**Model**	< 0.0001	0.0003	< 0.0001	< 0.0001	<0.0001
X_1	< 0.0001	< 0.0001	< 0.0001	< 0.0001	0.0002
X_2	0.1258	0.1800	0.0881	0.1268	<0.0001
X_1X_2	0.0097	0.0284	0.0483	0.1097	0.0007
X_1^2	0.0006	0.0071	0.0011	0.0039	0.0002
X_2^2	0.3417	0.2862			<0.0001
$X_1^2X_2$					0.0015
$X_1X_2^2$					0.0025
Lack of Fit	0.2569	0.1064	0.0895	0.1506	0.7573

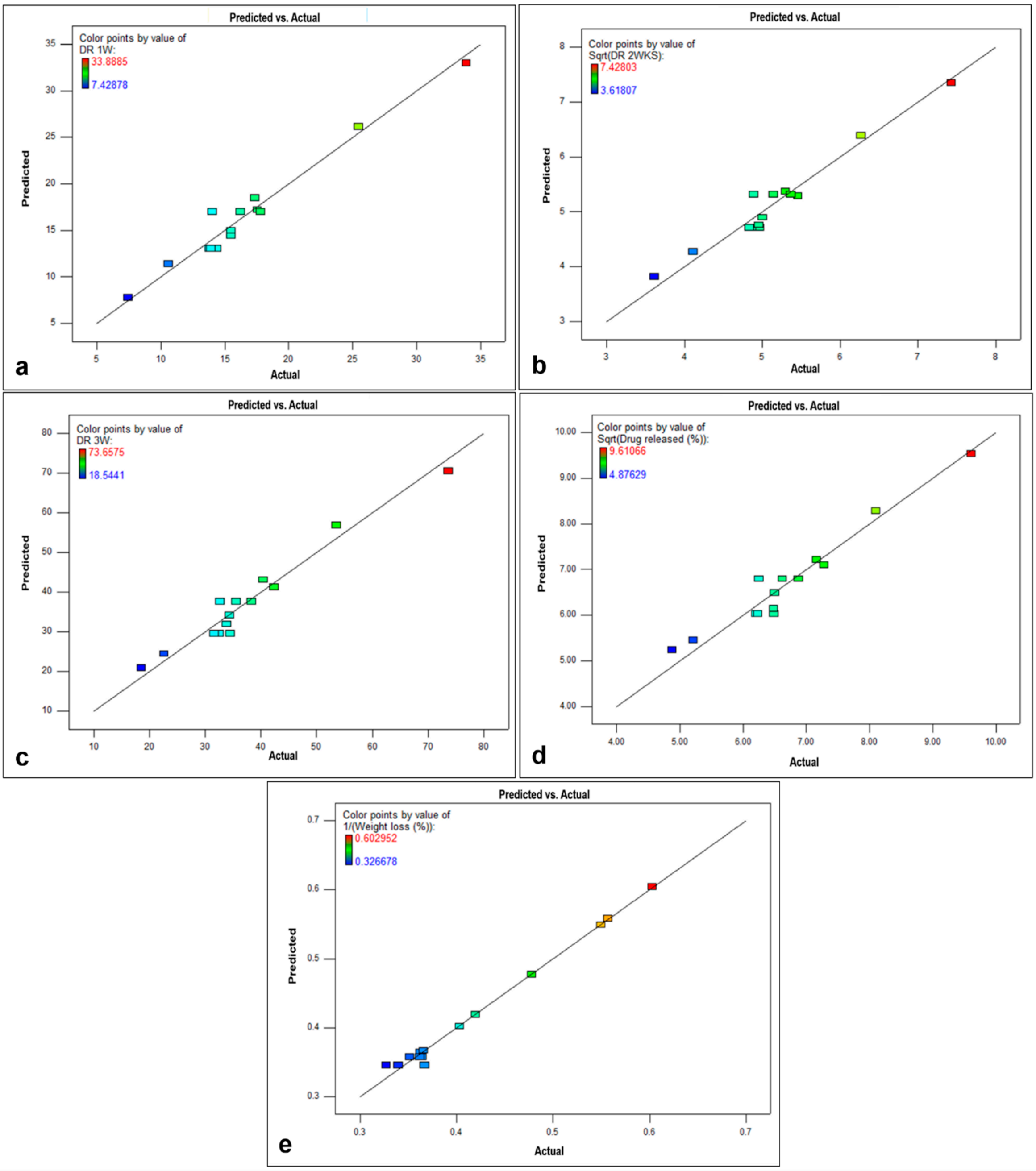

Figure S3. Plot of the measured and model-predicted values of the response (a) Y1, (b) Y2, (Y3), (Y4), and (Y5) of EPHCDs.

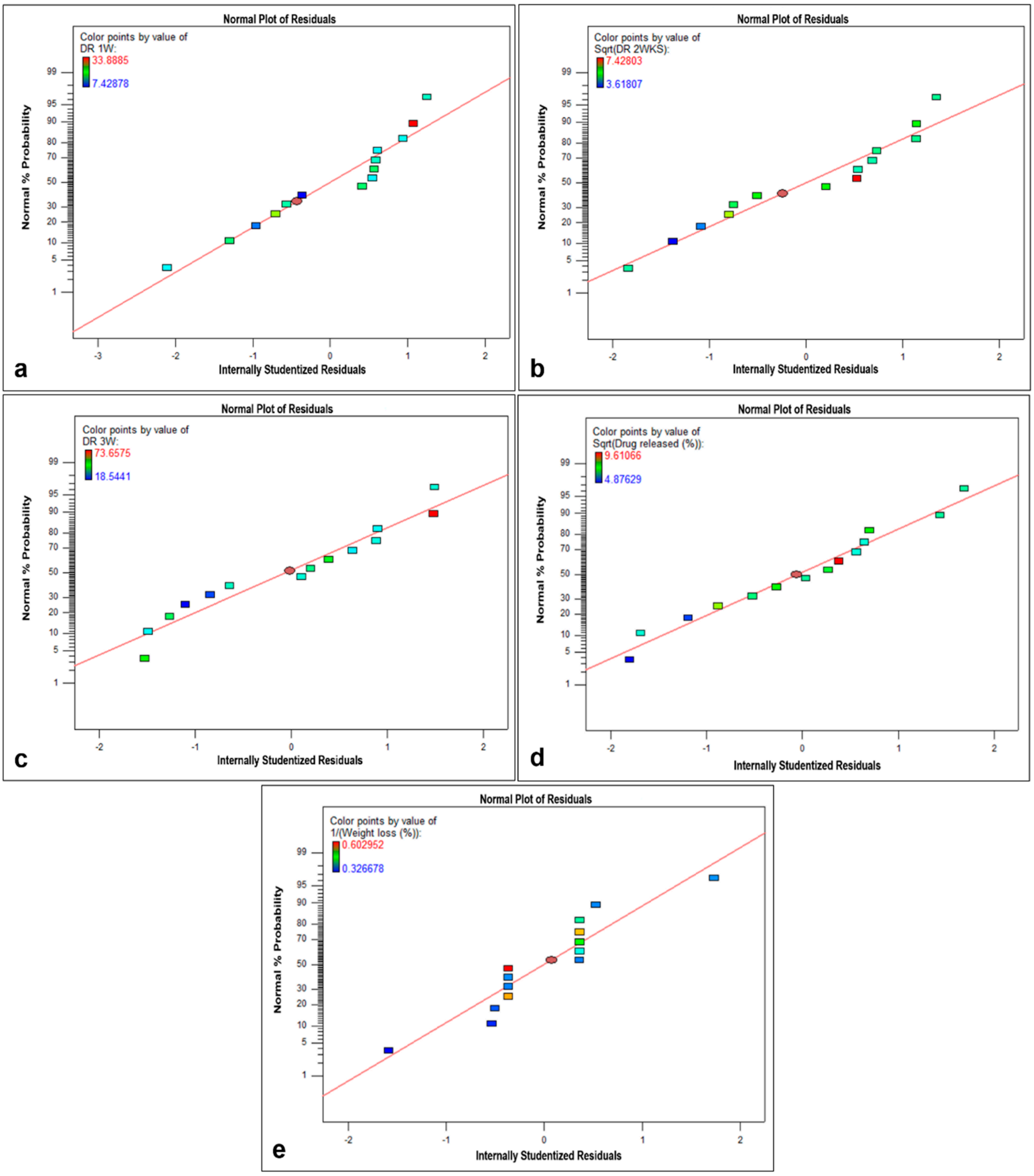

Figure S4. The normal probability plot of the residuals for (a) Y1, (b) Y2, (c), Y3 (d) Y4, and (e) Y5 of EPHCDs.

S.2. Constrained statistical Optimization of the EPHCD

The primary aim of employing the CCD-based optimization approach was to derive the optimal formulation parameters required for the preparation of the EPHCD. To achieve this goal, constraints were imposed on both the dependent and independent variables. A constraint referred to as 'in-range' was applied to independent variables, while the constraints imposed on the dependent variables, namely drug release and weight loss%, were set as to (minimize).

Table S2 showcasing the chosen numerical optimization solution. The table provides a comprehensive insight into the specific values and conditions that were identified as optimal for the study. Figure S5a represent the contour plot of the numerical optimization

illustrating the selected optimum solution. Moreover, Figure S5b portraying the constructed desirability landscape, an optimal design space, achieved by superimposing contour plots and main effect plots derived by using graphical optimization module of the implemented CCD. This graphical representation elucidated regions in the design space aligning to the specified optimization criteria, distinct in bright yellow. Consequently, the CCD model generated an optimized formulation, indicating that the formulation comprise a drug load of 10% and 10% ratio of EC to PCL. This optimized formulation was projected to yield the optimum results for both the 4-week percentage drug release and weight loss, achieving values of 31.7% and 2.07%, respectively.

Table S2. The selected optimal configuration from numerical optimization.

**Drug load (%)**	**EC to PCL ratio (%)**	**Drug released W1 (%)**	**Drug released W2 (%)**	**Drug released W3 (%)**	**Drug released W4 (%)**	**Weight loss (%)**	**Desirability**
**10**	10	9.71629	17.0243	24.9693	31.6712	2.07283	0.855

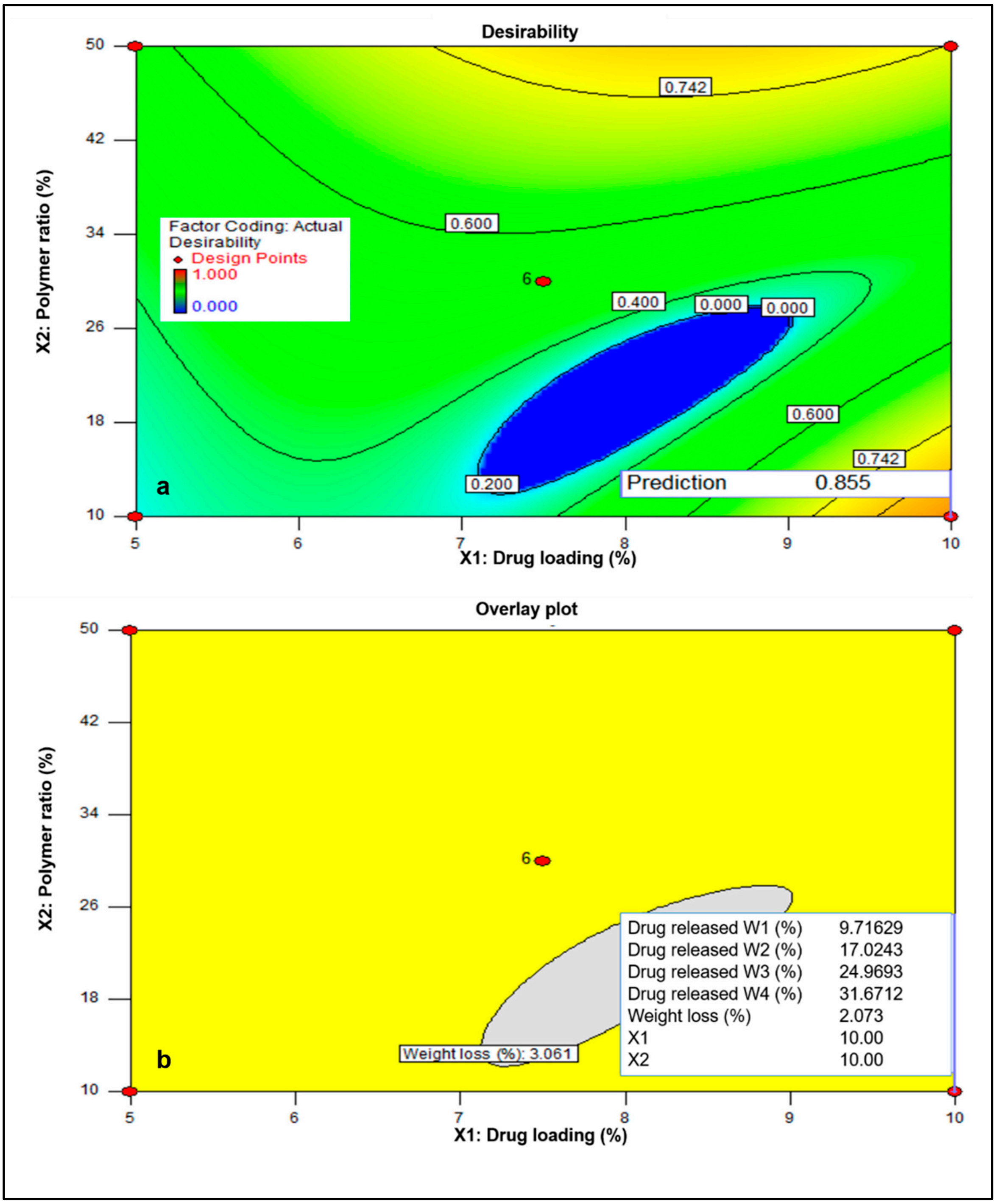

Figure S5. Contour plot of numerical optimization (a) and overlay plot of graphical optimization (b).

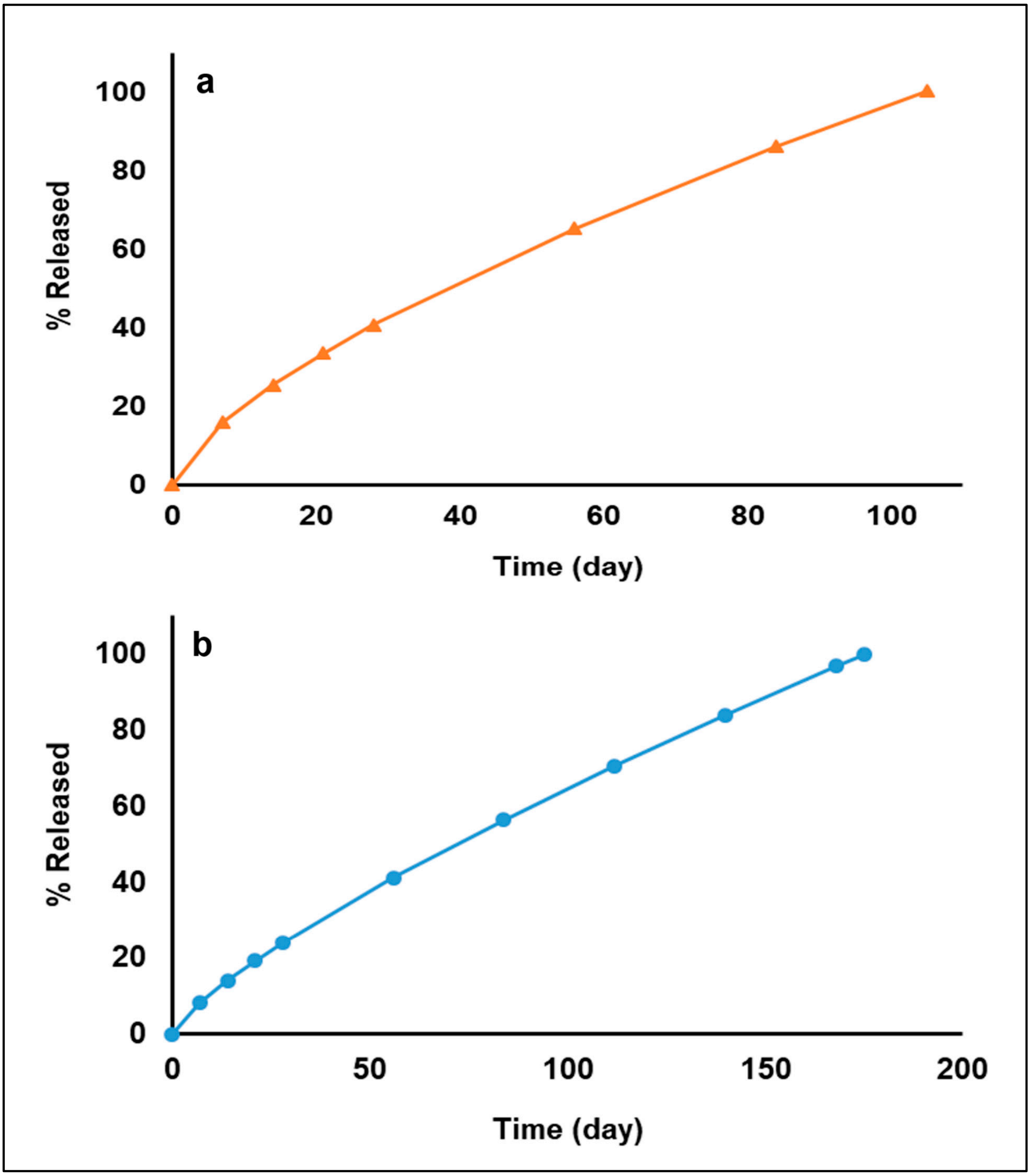

Figure S6. The expected release for (a) NETA and (b) E2 from MCDDS, using the Korsmeyer-Peppas model.

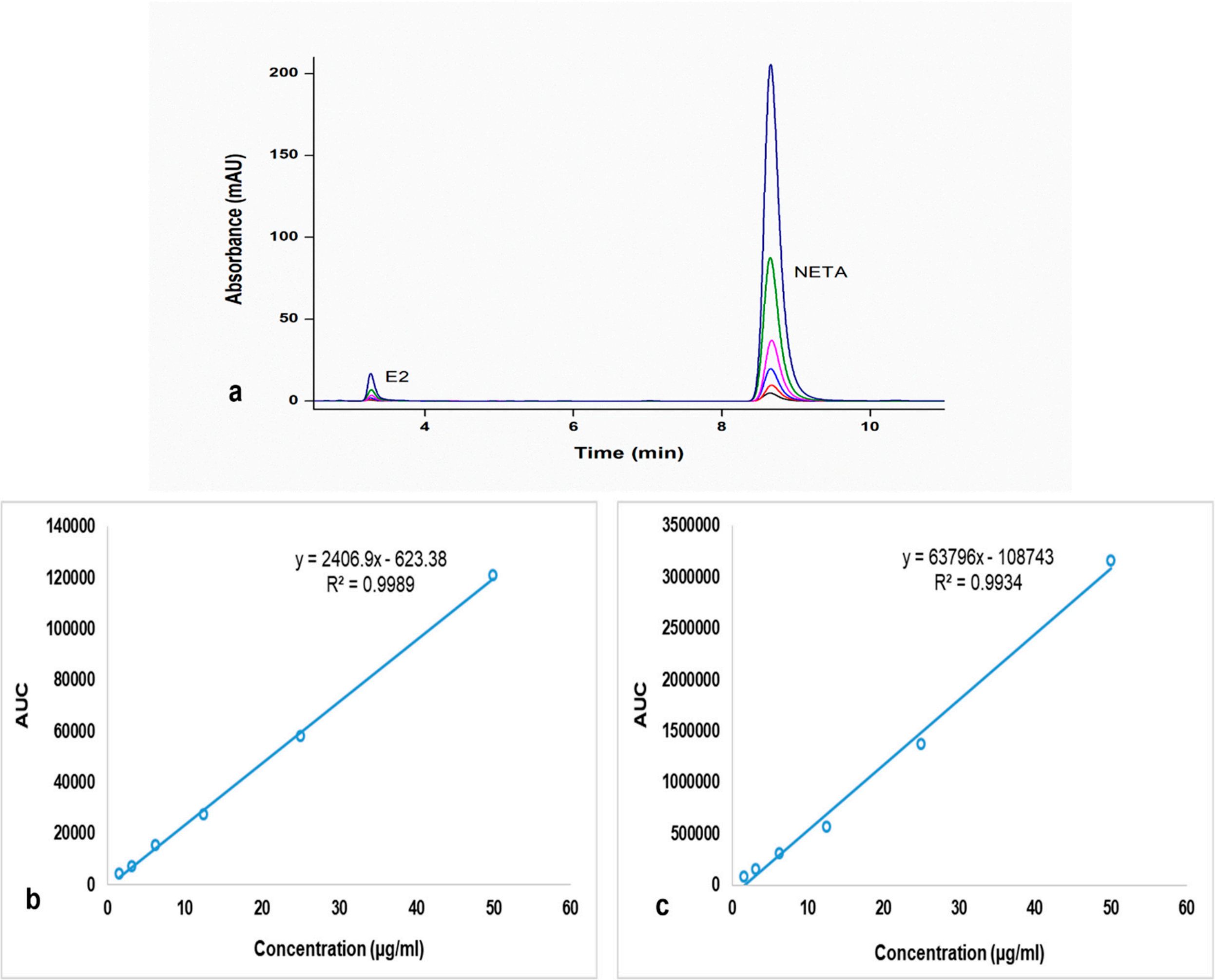

Figure S7. (a) HPLC chromatogram of E2 and NETA, (b) standard curve of E2, and (c) standard curve of NETA.
